# The landscape of regional missense mutational intolerance quantified from 125,748 exomes

**DOI:** 10.1101/2024.04.11.588920

**Published:** 2024-04-13

**Authors:** Katherine R. Chao, Lily Wang, Ruchit Panchal, Calwing Liao, Haneen Abderrazzaq, Robert Ye, Patrick Schultz, John Compitello, Riley H. Grant, Jack A. Kosmicki, Ben Weisburd, William Phu, Michael W. Wilson, Kristen M. Laricchia, Julia K. Goodrich, Daniel Goldstein, Jacqueline I. Goldstein, Christopher Vittal, Timothy Poterba, Samantha Baxter, Nicholas A. Watts, Matthew Solomonson, gnomAD Consortium, Grace Tiao, Heidi L. Rehm, Benjamin M. Neale, Michael E. Talkowski, Daniel G. MacArthur, Anne O’Donnell-Luria, Konrad J. Karczewski, Predrag Radivojac, Mark J. Daly, Kaitlin E. Samocha

**Affiliations:** 1 Program in Medical and Population Genetics, Broad Institute of MIT and Harvard, Cambridge, MA, USA; 2 Center for Genomic Medicine, Massachusetts General Hospital, Boston, MA, USA; 3 Bioinformatics and Integrative Genomics Program, Harvard Medical School, Boston, MA, USA; 4 Analytic and Translational Genetics Unit, Massachusetts General Hospital, Boston, MA, USA; 5 Stanley Center for Psychiatric Research, Broad Institute of MIT and Harvard, Cambridge, MA, USA; 6 Department of Medicine, Harvard Medical School, Boston, MA, USA; 7 Khoury College of Computer Sciences, Northeastern University, Boston, MA, USA; 8 Centre for Population Genomics, Garvan Institute of Medical Research and UNSW Sydney, Sydney, New South Wales, Australia; 9 Centre for Population Genomics, Murdoch Children’s Research Institute, Melbourne, Victoria, Australia; 10 Division of Genetics and Genomics, Boston Children’s Hospital, Boston, MA, USA; 11 Institute for Molecular Medicine Finland (FIMM), Helsinki, Finland

## Abstract

Missense variants can have a range of functional impacts depending on factors such as the specific amino acid substitution and location within the gene. To interpret their deleteriousness, studies have sought to identify regions within genes that are specifically intolerant of missense variation^1-12^. Here, we leverage the patterns of rare missense variation in 125,748 individuals in the Genome Aggregation Database (gnomAD)^13^ against a null mutational model to identify transcripts that display regional differences in missense constraint. Missense-depleted regions are enriched for ClinVar^14^ pathogenic variants, *de novo* missense variants from individuals with neurodevelopmental disorders (NDDs)^15,16^, and complex trait heritability. Following ClinGen calibration recommendations for the ACMG/AMP guidelines, we establish that regions with less than 20% of their expected missense variation achieve moderate support for pathogenicity. We create a missense deleteriousness metric (MPC) that incorporates regional constraint and outperforms other deleteriousness scores at stratifying case and control *de novo* missense variation, with a strong enrichment in NDDs. These results provide additional tools to aid in missense variant interpretation.

## Main text

Over the last decade, exome and genome sequencing have enabled variant discovery across hundreds of thousands of individuals^13,17-21^. These large reference databases have provided the opportunity to study selective forces acting on the human genome and to identify genomic regions under selective constraint by, for example, identifying regions with fewer variants than expected based on mutational models^13,18,22-25^. Gene-level metrics of predicted loss-of-function (pLoF) variant depletion have proven to be valuable in variant classification and identification of novel disease genes^15,16,26-28^. The functional impact and selective pressures relevant to missense variation, by contrast, remain challenging to predict, as the effect of a missense variant is governed by the gene housing the variant, the position of the variant in the gene, and the specific amino acid substitution caused by the variant. To address this, prior work has sought to identify regions within coding genes that are specifically intolerant of missense variation as a way to improve interpretation^1-12^. Here, we expand upon previous work^1^ and show a sub-genic measure of missense intolerance leveraging population-level variation facilitates variant classification and risk stratification for association studies with *de novo*, rare, and common variants.

**Fig. 1:**
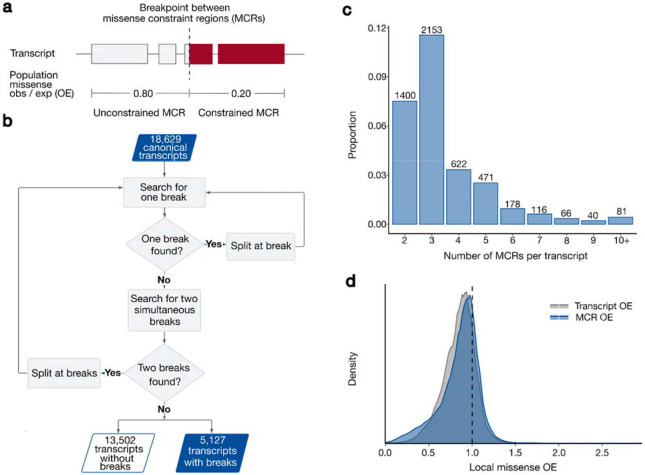
28% of protein-coding genes in the human genome are discovered to harbor regional variation in population-level missense depletion. **a**, An example transcript that has two missense constraint regions (MCRs) with significantly different levels of population-wide missense depletion, defined as the number of missense variants observed in gnomAD at rare frequency (AF < 0.1%) divided by the number of rare missense variants expected under neutral evolution (observed/expected or OE). Lower OE values correspond to greater variant depletion in the population and suggest stronger constraint. **b**, Flow chart describing the process of searching for breakpoints that divide a transcript into multiple MCRs. Searching for breakpoints is recursive and leverages likelihood ratio tests at a significance threshold of p = 0.001. **c**, The number of MCRs within the 5,127 transcripts discovered to harbor regional differences in missense constraint. The other 13,502 transcripts are deemed to have a single MCR (that is, a constant level of constraint across their entirety) and are not shown. **d**, The distribution of local missense OE at all coding sites in canonical transcripts. Local missense OE is defined as the OE calculated over the whole transcript (for “transcript OE”) or over the MCR (for “MCR OE”) where the site is located. Transcript OE and MCR OE are equivalent for transcripts with one MCR.

We explored the patterns of rare missense variant presence or absence in 125,748 exomes in the Genome Aggregation Database (gnomAD) v2.1.1 on GRCh37 to quantify missense depletion at the sub-genic level. We searched 18,629 canonical protein-coding transcripts for variability in missense constraint, quantified as the number of rare (allele frequency [AF] < 0.1%) missense variants observed in gnomAD divided by the number expected under neutral evolution as estimated from previously described mutational models^13^(observed/expected [OE]). For each transcript, we applied a recursive search based on likelihood ratio tests over all potential rare missense sites looking for breaks that divide the transcript coding sequence (CDS) into distinct missense constraint regions (MCRs; Fig. 1a, b). We discover 5,127 transcripts (28%) harbor regional variability in missense constraint (Fig. 1c), i.e., have two or more MCRs (minimum coding length 49bp, median 461bp; Supplementary Fig. 1). We thus refine the resolution of missense constraint for 42% of coding sites (coding space in the 5,127 transcripts vs. 18,629 total assessed). After recalibrating the missense OE distribution over all potential sites of missense variants using MCR-wide rather than transcript-wide missense OE measurements, we discover widespread signatures of negative and neutral selection that are obscured when quantifying over the unit of whole transcripts (Fig. 1d). We find a larger proportion of the exome lies within strongly constrained sequences (5.6% vs. 1.7% at OE < 0.4; see Supplementary Note for OE threshold selection), and the mode of the distribution shifts toward an OE indicative of evolutionary neutrality at approximately 1 (40.6% vs. 36.5% at 0.9 < OE ≤ 1.1).

**Fig. 2:**
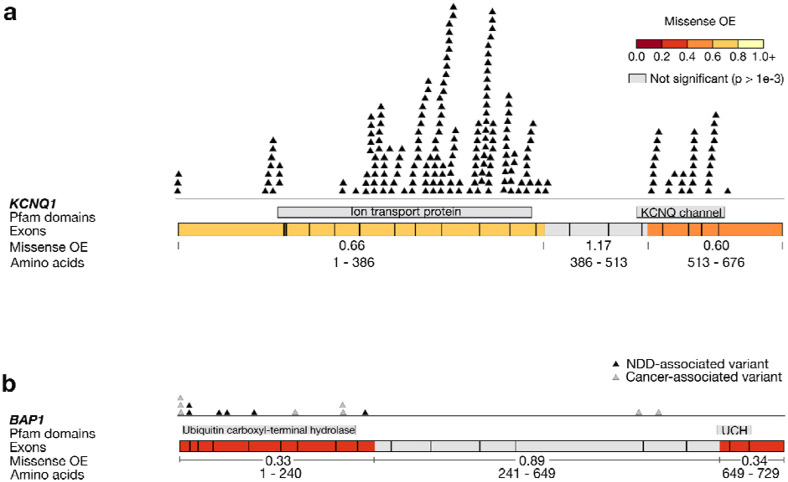
Missense constraint regions (MCRs) and the distribution of ClinVar pathogenic/likely pathogenic (P/LP) missense variants in two genes associated with early-onset developmental disorders. Exons are delineated with black outlines and MCRs are delineated by color. MCRs are colored based on their missense observed/expected (OE) ratio, and MCRs with missense OEs not significantly different from 1 (p > 0.001) are shaded gray. **a,** *KCNQ1*. Only two of the 210 P/LP missense variants in *KCNQ1* do not fall within either constrained MCR. The first constrained MCR encompasses the voltage-sensing and pore domains of this gene, and the other constrained MCR overlaps the C-terminus. Both domains contain previously reported hotspot regions, with some regions reaching moderate level (PM1) support for pathogenicity^31^. Ion transport protein: domain that contains both the transmembrane voltage-sensing and pore domains. KCNQ channel: C-terminal cytoplasmic domain that overlaps four helices (A-D). **b,** *BAP1*. Variants in this gene can lead to cancer-predisposition syndromes, increased risk of certain cancers, or the neurodevelopmental disorder Kury-Isidor syndrome^32^. All of the ClinVar P/LP variants associated with Kury-Isidor fall within the first MCR with a highly depleted missense OE of 0.33. An additional five variants reported in Kury *et al*.^32^ but not ClinVar fall within either highly constrained MCR in this gene. P/LP variants associated with Kury-Isidor are colored in black, and all other cancer-associated P/LP variants are colored in gray. UCH: Ubiquitin carboxyl-terminal hydrolase isozyme L5 domain. ClinVar data are from the October 15, 2023 release.

Furthermore, we find that constrained MCRs overlap established disease-associated mutational hotspots, including critical protein domains. One example is in the well-characterized *KCNQ1*, a voltage-gated potassium channel gene, in which pathogenic variants cause cardiac disorders such as long QT syndrome. We discover one moderately constrained MCR (missense OE = 0.60) overlapping the highly conserved C-terminus ^29^ and another (missense OE = 0.66) encompassing the voltage-sensing and pore domains (Fig. 2a). Both the C-terminus of *KCNQ1* and its voltage-sensing domain are established “hotspot” regions (specific missense-constrained regions with ACMG/AMP hotspot/functional domain moderate support [PM1] for pathogenicity)^29-31^. All but two ClinVar pathogenic/likely pathogenic (P/LP) missense variants in this gene fall within these two missense-constrained MCRs.

We also find that missense constraint within MCRs is able to identify regions associated with severe, early-onset disease. One example is in *BAP1*, which plays a key role in chromatin modeling by mediating histone deubiquitination. Disease-causing variants in this gene are linked to cancer or, as recently discovered, Kury-Isidor syndrome^32^. The first highly missense-constrained MCR (missense OE = 0.33) in *BAP1* encompasses the ubiquitin C-terminal hydrolase domain connected to Kury-Isidor syndrome^32^ (Fig. 2b), and all 11 variants reported to be causal for Kury-Isidor fall within this MCR. The only ClinVar P/LP variants that do not fall within any missense-constrained MCRs in *BAP1* are associated with cancer phenotypes, which may be under weaker selection than neurodevelopmental disorders (NDDs).

**Fig. 3:**
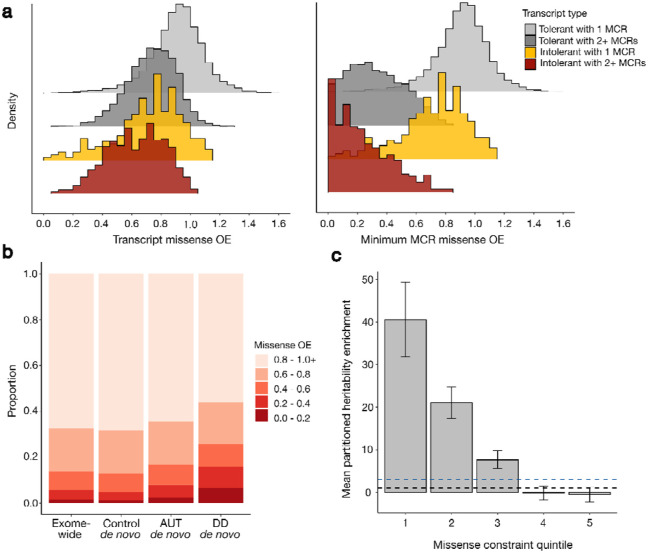
Regional missense depletion reveals constraint obscured by gene-level measures. **a,** Left: The distribution of transcript-wide missense observed/expected (OE) across 18,629 transcripts stratified by the combination of two factors: whether the transcript is strongly mutationally intolerant (within first three LOEUF deciles and association with a developmental phenotype in Gene2Phenotype [G2P]^34^) and whether we detect multiple missense constraint regions (MCRs). Number of transcripts in each category are: strongly intolerant with multiple MCRs (n=581; red), strongly intolerant with one MCR (n=149; yellow), not strongly intolerant with multiple MCRs (n=4,546; dark gray), not strongly intolerant with one MCR (n=13,353; light gray). X-axis is cut off at 1.6 for visibility. Right: Minimum MCR missense OE using the same groupings. Minimum MCR missense OE is the same as transcript missense OE for transcripts with a single MCR. **b**, MCR missense OE at all sites of possible exome-wide missense variants vs. sites of *de novo* missense variants in controls, autistic individuals (AUT), or individuals with DD. *De novo* variants from individuals with developmental phenotypes are enriched in more constrained sequences, with a more pronounced enrichment in DD than autism. **c**, Enrichment in per-variant heritability explained by common (AF > 5%) protein-coding SNPs stratified by MCR missense OE quintile, relative to the average SNP genome-wide. Enrichment is estimated by linkage disequilibrium score regression, accounting for number of SNPs in each quintile, and is averaged across 268 independent traits 198 in UKBB and other large genome-wide association studies. Black dashed line at 1 indicates no enrichment. Blue dashed line at 3 indicates average coding enrichment. Error bars represent 95% confidence intervals.

Next, we sought to determine if the signatures of selection revealed by MCRs recapitulated biological and disease relevance of coding sequences. Overall, most transcripts that are intolerant to pLoF variation (as measured by the loss-of-function observed/expected upper bound fraction [LOEUF] score^13^) also tend to be intolerant to missense variation. This trend is markedly more prominent when measuring missense constraint at the sub-genic level vs. the transcript-level (Supplementary Note; Supplementary Fig. 2). We also discovered that 64% (1697/2659) of genes that are both LOEUF- and MCR missense-constrained do not have disease associations in OMIM^33^, suggesting the existence of many undocumented genes containing variants of significant consequence for disease (Supplementary Fig. 3). In a set of 730 strongly mutationally intolerant genes, defined here as exhibiting both population depletion of pLoF variants (first three LOEUF deciles) and association with a developmental phenotype (high-confidence membership in any non-cancer Gene2Phenotype [G2P]^34^ gene list with dominant inheritance), we observed strong transcript-wide missense depletion that was even stronger for genes with multiple MCRs (Fig. 3a and Supplementary Fig. 4; Wilcoxon p < 10^−50^). Given that we have greater power to detect missense constraint variability over longer sequences (Supplementary Fig. 5), we controlled for transcript length but still found that intolerant transcripts are eight times more likely to harbor multiple MCRs (p < 10^−50^). These strongly intolerant transcripts are highly enriched for severely depleted regions (three times more likely to have minimum MCR OE < 0.4 after regressing out transcript length, p < 10^−18^), whereas the most constrained MCRs in not strongly intolerant transcripts are less depleted and more evenly distributed across the OE spectrum. Finally, we observe a group of genes with strong overall missense depletion in which we did not detect multiple MCRs (n = 459 with missense OE < 0.4; Supplementary Table 1), suggesting these genes are robustly intolerant to missense variants across their length. When comparing missense constraint to selection over longer timescales (measured by evolutionary conservation in placental mammals, phyloP^35^), we found that genes with more conserved coding sequences also tended to be more overall depleted of human missense variation (Spearman ρ = 0.56, p < 10^−50^). However, a substantial number of strongly constrained MCRs appear widely unconserved across mammals, potentially pointing to human-specific negative selection pressures that are obscured at the whole-transcript level (Supplementary Fig. 6).

We next aggregated *de novo* missense variants from 31,058 individuals with a severe developmental disorder^15^ (DD), 15,036 autistic individuals (AUT), and 5,492 siblings not diagnosed with a DD^16^ (Fig. 3b). The distribution of *de novo* missense variants across the missense OE spectrum in unaffected siblings largely mirrored the exome-wide missense OE distribution. In contrast, *de novo* missense variants in autistic individuals are enriched in missense-constrained sequences, and this pattern is more striking in individuals with DDs. For example, relative to unaffected siblings, the rate of *de novo* missense variants in MCRs with OE < 0.2 is 2-fold higher in autistic individuals (p < 10^−23^) and 6.6-fold higher in individuals with DDs (p < 10^−50^) (Supplementary Fig. 7; see Supplementary Note for OE threshold selection). This is consistent with the expectations that a small subset of *de novo* missense variants in individuals with developmental phenotypes are causal for those traits and that variants causal for DD are generally more selectively deleterious than those for autism.

Beyond large-effect rare and *de novo* variation in traits under strong negative selection, we additionally investigated whether our MCR metric, which was calculated using rare variants, correlates with functional effects of common variants. Prior work found that pLoF-constrained genes and their flanking 100kb sequences are enriched for SNP heritability across hundreds of independent traits in the UK Biobank (UKBB) and other large genome-wide association studies (GWAS) ^13^. We partitioned common (AF > 5%) variant heritability of the same 268 independent traits across MCRs to investigate relative enrichment. To establish a baseline, we computed the heritability enrichment over all coding sequences comprising MCRs (3-fold). The most constrained MCRs have the strongest heritability enrichment; the first quintile of MCR missense OE harbors a 41-fold enrichment (Fig. 3c). Coding SNPs in missense-unconstrained MCRs (e.g., in the two least constrained quintiles of MCR missense OE) harbor no detectable heritability enrichment relative to the average genome-wide SNP. These findings suggest that: 1) regions depleted of rare missense variation can help prioritize common coding variants important for complex traits (i.e., improve GWAS fine-mapping variant prioritization), and 2) there exists a subset of coding sequence with no appreciable heritability enrichment, which rare variant depletion can help identify.

**Fig. 4:**
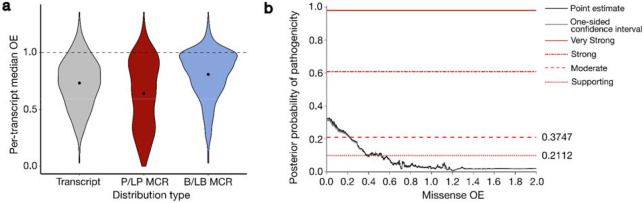
ACMG/AMP calibration of missense constraint. **a,** The distribution within genes with autosomal dominant disease associations of transcript-wide missense observed/expected (OE; gray) and missense constraint region (MCR) OE for ClinVar pathogenic/likely pathogenic missense variants (P/LP; red) and benign/likely benign missense variants (B/LB; blue). We filtered to 1,007 transcripts with at least one P/LP and one B/LB missense variant. For the P/LP and B/LB distributions, we annotated each variant with the missense OE across the MCR they fell in and collapsed these values within each transcript by taking the respective medians. **b,** Local posterior probabilities of pathogenicity given MCR missense OE in all transcripts. Gray shading indicates the one-sided 95% confidence interval on the more stringent side. Horizontal lines indicate thresholds required to meet ACMG/AMP evidence levels. From bottom to top: supporting, moderate, strong, very strong. MCR missense OE reaches supporting (OE ≤ 0.37) and moderate (OE ≤ 0.21) level evidence for PM1 (hotspot/functional domain).

We examined the localization of high-quality ClinVar^36^ missense variants classified as P/LP within genes with both unconstrained (missense OE > 0.9) and constrained (missense OE < 0.2) MCRs and found that P/LP variants occur much more frequently in missense constrained MCRs (odds ratio [OR] = 15.2; p < 10^−50^). We also examined the localization of P/LP and benign/likely benign (B/LB) variants within MCRs in autosomal dominant disease-associated genes and found that P/LP variants tend to localize to regions that are more strongly missense-constrained than the overall transcript (Wilcoxon p = 3.5x10^−10^), while B/LB variants show the opposite effect and tend to occur in regions with OEs closer to 1 (Wilcoxon p < 10^−18^; Fig. 4a). While more subtle, these same patterns are also significant in autosomal recessive disease-associated genes (Supplementary Fig. 8).

To enable use of our missense constraint metric in ACMG/AMP clinical variant classification, we applied previously established probabilistic frameworks^37^ to determine the MCR missense OE thresholds that met different levels of clinical evidence strengths evaluated under the hotspot/functional domain (PM1) and benign *in silico* prediction (BP4) criteria codes^30^. MCR missense OE ≤ 0.37 met supporting (PM1_Supporting) and OE ≤ 0.21 met moderate (PM1) levels of evidence for pathogenicity (Fig. 4b), but no MCR missense OE threshold met any levels of evidence to support benignity. However, separate calibration specifically in transcripts with multiple MCRs found that MCR missense OE ≥ 1.56 met moderate and OE ≥ 0.97 met supporting evidence for BP4, indicating that in transcripts where we are powered to characterize regional constraint, MCRs with OEs close to one harbor an indication of benignity (Supplementary Fig. 9). Calibration of two additional regional constraint metrics, Constrained Coding Regions (CCRs^9^) and COntact Set MISsense tolerance (COSMIS^12^), which incorporates predicted 3D structure information, revealed that these metrics also reach moderate support for pathogenicity (PM1), and COSMIS only reaches supporting levels for benignity (Supplementary Fig. 9; Supplementary Table 2).

**Fig. 5:**
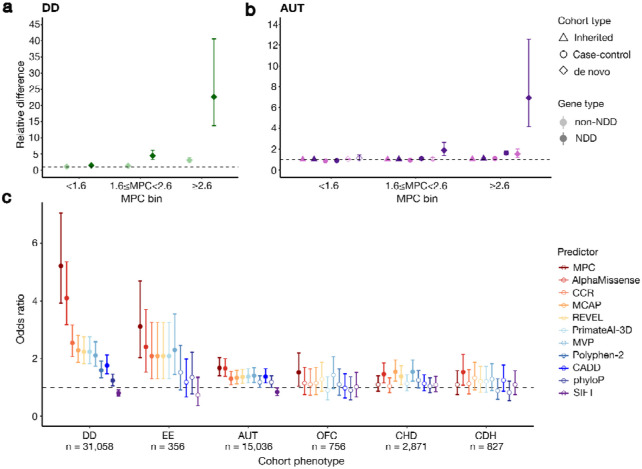
MPC effectively stratifies case and control variation. **a,** The difference relative to controls of missense variants stratified by MPC score and localization to genes associated with neurodevelopmental disorders (NDDs) for **a,** individuals with DD and **b,** autistic individuals (AUT). Relative difference is calculated as: for *de novo* variants, the average rate of variants in probands divided by that in sibling controls; for case-control, the average rate of variants in cases divided by that in controls from case-control data; for inherited, the average rate in probands of transmitted variants divided by that of untransmitted variants. Error bars represent 95% confidence intervals calculated from a binomial test. **c,** The odds ratio of case to control *de novo* missense variants in the top 10% vs. bottom 90% of respective rankings. *De novo* missense variants from each case cohort are ranked against those in the 5,492 controls for each predictor. DD: developmental disorders, EE: epileptic encephalopathy, AUT: autism, OFC: orofacial cleft, CHD: congenital heart disease, CDH: congenital diaphragmatic hernia. Error bars represent 95% confidence intervals. Only variants scored by all predictors are included. Points are solid colored if the difference from 1 is statistically significant (binomial or Fisher exact p < 0.05).

We transformed our regional missense constraint measure into a variant-level predictor of missense deleteriousness named MPC (Missense deleteriousness Prediction by Constraint) that additionally incorporates information about amino acid substitution type and local context. The logistic regression-based model integrates regional missense constraint-derived metrics together with BLOSUM^38^, Grantham^39^, and PolyPhen-2^40^ and is trained on ClinVar pathogenic and gnomAD common (AF > 0.1%) variants in 2,987 genes defined as haploinsufficient in Collins *et al*.^41^ and 366 genes with DD associations in G2P through non-LoF mechanisms. Higher scores predict greater deleteriousness (Supplementary Fig. 10, 11). We assessed the utility of MPC in prioritizing potentially disease-causing variation by evaluating its ability to stratify case and control rare and *de novo* missense variation. Consistent with the regional constraint results, the *de novo* missense variants from DD and AUT cases are enriched for high MPC scores compared to controls (Supplementary Fig. 12). We further stratified by presence in 373 genes previously associated with NDD^16^ and three bins of MPC scores (< 1.6, 1.6-2.6, ≥ 2.6; see Supplementary Note for calibration of these bins), and found a very strong enrichment of *de novo* missense variants in the two most deleterious bins among both the DD (Fig. 5a) and AUT cases (Fig. 5b) compared to unaffected individuals. However, while the enrichment in the 373 NDD-associated genes was significant for missense variants with MPC ≥ 2.6 (RR in DD cases = 22.7, p < 10^−50^; RR in AUT cases = 6.9, p < 10^−21^), as well as missense variants with MPC between 1.6-2.6 (RR in DD cases = 4.5, p < 10^−35^; RR in AUT cases = 1.9, p = 3.0x10^−5^), it was only significant in NDD-unassociated genes for missense variants with MPC ≥ 2.6 (RR in DD cases = 3.1, p < 10^−28^; RR in AUT cases = 1.5, p = 5.9x10^−4^). This suggests that while there is a sizable reservoir of potentially causal variants in genes yet to be associated with NDDs, they will be more difficult to find as they must reach stricter deleteriousness criteria. For autism, we additionally assessed inheritance rates of rare missense variants (AF < 0.1%) from parents to 13,384 probands and case-control rates for an additional 5,591 cases and 8,597 controls without *de novo* information. While we did not find substantial enrichment in inheritance rates in any missense category, we discovered substantial enrichment in the case-control analysis for variants in the 373 NDD-associated genes with MPC ≥ 2.6 (RR = 1.6, p < 10^−12^), which we infer is from *de novo* variants that are not recognizable as such due to lack of parental information.

We extended our assessment of case-control *de novo* stratification for a comparison of our model against several other missense deleteriousness predictors: AlphaMissense^42^, CCRs^9^, M-CAP^43^, REVEL^44^, PrimateAI-3D^45^, MVP^46^, Polyphen-2^40^, CADD^47,48^, mammalian conservation phyloP^35^, and SIFT^49^. For this assessment, we evaluated four additional early-onset development-related phenotypes: epileptic encephalopathy (EE), orofacial cleft (OFC), congenital heart disease (CHD), and congenital diaphragmatic hernia (CDH). To compare across predictors with different score distributions, we used a ranking-based performance assessment. For each predictor, we ranked the *de novo* missense variants from each case cohort against those in the 5,492 controls and computed the OR of case vs. control variants in the top percentiles of these rankings (Fig. 5c). At the top 10% of variants, MPC displays the highest OR for DD (OR = 5.2, Fisher’s exact p < 10^−48^), EE (OR = 3.1, p = 2.2x10^−7^), AUT (OR = 1.7, p = 8.9x10^−9^), and OFC (OR = 1.5, p = 0.025), although there is substantial confidence interval overlap with other predictors. This indicates that MPC effectively ranks high-impact *de novo* variants in the most deleterious prediction regimes. Of the other predictors, AlphaMissense also performs consistently well across all phenotypes. In particular, in CHD and CDH, which have the least *de novo* enrichment across predictors, we observe MPC lagging in performance, while AlphaMissense is one of the top performers. This may suggest that causal *de novo* variants in these phenotypes may occur at a narrow set of sites where 3D structure is important, which AlphaMissense can more deftly capture through integration of protein structure prediction. These observations are more or less consistent over a range of thresholds used to define the ranking top percentiles (Supplementary Fig. 13).

We have developed a method to identify sub-genic regions with differential intolerance to missense variation at base-level resolution. We demonstrate that coding regions depleted for missense variation in the general population are enriched for established disease-associated variation, *de novo* variants from individuals with NDDs, and heritability for 268 complex traits from the UK Biobank and other large GWAS. Additionally, we have calibrated these constraint scores to establish that regions with less than 20% of their expected variation can achieve moderate evidence for association to disease following ACMG/AMP guidelines. Finally, we incorporated regional missense intolerance information into the missense deleteriousness metric, MPC, and show that MPC effectively separates potentially risk-carrying variants identified in various developmental disorder cases from those seen in controls.

At current sample sizes, we are unable to characterize constraint at single amino acid resolution. Furthermore, because our approach relies on variant presence or absence in a large reference dataset, many of the constrained regions we find are linked to variants that cause severe, early-onset disease. However, the true nature of the variation we capture is more accurately linked to reproductive fitness and the strength of selection acting on heterozygotes^50^. Our methodology specifically searches for linear sub-genic regions in canonical transcripts that are depleted of missense variants compared to a null mutational model. This means that our model is unable to find depleted sequences that are clustered specifically in 3D space and is also currently ignorant of coding sequences not present in the Ensembl canonical transcript. However, we note that our linear metric achieves similar evidence for both pathogenicity and benignity as the structural constraint-based COSMIS model^12^ (Supplementary Fig. 9).

In summary, we identify 28% of canonical transcripts with variable levels of missense constraint and demonstrate that coding regions specifically depleted of missense variation in the general population are enriched for disease-associated variation. Additionally, we show that this depletion of missense variation can be used as moderate evidence when classifying variants according to ACMG/AMP guidelines and that incorporation of regional missense constraint into an *in silico* predictor effectively prioritizes a subset of *de novo* missense variation in individuals with developmental phenotypes for association testing. We have publicly released these data for use in both research and clinical settings. We anticipate refined resolution of these metrics as datasets grow, both in size and in ancestral diversity, and with the incorporation of complementary structural or functional data.

## Methods

### Transcripts

This study analyzed only canonical, coding transcripts as defined by GENCODE v19/Ensembl v74. We excluded the same set of transcripts from this analysis that were excluded in the previous gnomAD v2.1.1 genic constraint estimates^13^. Briefly, we excluded transcripts that had outlier counts of variants expected under neutrality (zero expected pLoF, missense, or synonymous variants; too many observed pLoF, missense, or synonymous variants compared to expectation; or too few observed synonymous variants compared to expectation). In total, this study analyzed 18,629 transcripts.

### gnomAD variants

All analyses in this paper were conducted using the 125,748 gnomAD v2.1.1 exomes^13^ on GRCh37. Median coverage was calculated on a random subset of the gnomAD exomes as described previously^13^. We defined the set of sites with possible missense variants using a synthetic Hail Table (HT) containing all possible single nucleotide variants in the exome. We annotated this HT with the Variant Effect Predictor (VEP, version 85) against GENCODE version 19, and filtered to variants with the consequence "missense_variant" in the canonical, coding transcripts as defined in *Transcripts*. We then further filtered to variants that fit one of the following criteria: (1) allele count (AC) > 0 and AF < 0.001, variant QC PASS, and median coverage > 0 in gnomAD v2.1.1 exomes; or (2) AC = 0, i.e. variants not seen in gnomAD v2.1.1 exomes.

### ClinVar variants

We annotated functional consequences for ClinVar^14^ (v.20230305) variants using the VEP table described in gnomAD variants. Missense ClinVar variants with non-conflicting P, LP, B, LB classification and a review status of at least one star were selected for analysis.

### Rare and *de novo* variants from developmental cohorts

Case *de novo* mutations for association analyses were obtained from studies of developmental disorders^15^ (DD), autism^16^ (AUT), congenital heart disease^51^ (CHD), orofacial cleft^52^ (OFC), congenital diaphragmatic hernia^53^ (CDH), and epileptic encephalopathy^54^ (EE). Control *de novo* mutations were obtained from neurotypical siblings of the autistic probands^16^. Variants from the autism study were lifted over from GRCh38 to GRCh37 using the “liftover” function in Hail. Variant functional consequences were re-annotated using the VEP table described in gnomAD variants. Variants transmitted and not transmitted from parents to autistic probands were procured from previously published ASC-SSC and SPARK cohorts, and case-control variants for autism were procured from previously published iPSYCH and Swedish cohorts^16^. Both the inherited/uninherited and case-control variant sets were filtered to AF < 0.1%.

### Training, validation, and test datasets

To generate independent training and test sets, we selected 80% (14,894 transcripts) of the 18,629 canonical coding transcripts to comprise the training set and the remaining 20% (3,735 transcripts) to the test set. To ensure the training and test transcripts have similar distributions of features that may impact constraint estimates, we used stratified randomization to match the training and test transcripts on *s*_*het*_ coefficients (as a measure of selection) and number of potential missense sites (as a measure of power to detect transcript-wide constraint changes). The training set was used for MPC model training and MCR model selection, and the test set was used for MPC model evaluation. No similar hold-outs of data were performed for training of the mutational model used to compute expected variant counts (see Modeling of mutation rates and expected neutral missense variation).

### Modeling of mutation rates and expected neutral missense variation

Expected missense variant counts were determined as described previously^13^. Briefly, we created a model using the 15,708 gnomAD v2.1.1 genomes that estimated the mutation rate for each single nucleotide substitution with one base of context (e.g., ACT > AGT) in non-coding regions of the genome. We then calibrated this mutation rate against the proportion observed of each context at synonymous sites to adjust for the larger size of the gnomAD v2 exomes, adjusting for low coverage regions (median coverage < 40x) and methylation levels at CpG sites using methylation data from the Roadmap Epigenomics Consortium^55^. We created three separate models (referred to as "plateau" models moving forwards): one for autosomal and pseudoautosomal sites, one for chromosome X sites, and one for chromosome Y sites. Each of these models contains mutation rate estimates for each substitution, context, and methylation level. We then applied the plateau models to the proportion observed of each substitution and its context, exome coverage, and methylation level. We counted all possible variants in our synthetic Hail Table (HT) that passed the following criteria: (1) Median coverage > 0; (2) no low-quality variant observed in gnomAD v2 exomes; (3) no variants above 0.1% AF observed in gnomAD v2 exomes. We then correlated this proportion observed value with the mutation rate calculated using the appropriate model above. For low coverage sites (median coverage below 40x), we calculated a scaling factor as described previously^13^: briefly, we computed the total number of observed synonymous variants in the gnomAD v2 exomes divided by the total number of possible synonymous variants in the synthetic HT multiplied by the mutation rate aggregated across all possible substitutions and their contexts and methylation levels. We used this scaling factor to create a model to adjust the proportion of expected variation for low coverage sites (coverage model).

### Identifying breakpoints within transcripts of regional missense constraint

Observed missense variant counts were calculated using sites from the 125,748 gnomAD exomes that passed all the following criteria: (1) Allele count (AC) > 0; (2) allele frequency (AF) < 0.001; (3) variant QC PASS (passed gnomAD variant QC filters, including random forest filters); (4) median coverage > 0. We filtered the gnomAD v2 exomes Hail Table (HT) to the sites that matched the above criteria and then annotated the synthetic HT with whether that variant (chromosome/locus plus reference and alternate alleles) was observed in the gnomAD exomes. We then aggregated the total number of observed variant counts per locus by summing the number of observed variants for each possible substitution (reference and alternate allele) at each locus. Finally, we grouped the synthetic HT by transcript annotation to sum the total number of observed missense variants per transcript.

As previously described^13^, we applied the two models (plateau and coverage) described in Modeling of mutation rates and expected neutral missense variation to calculate the total proportion of expected missense variation. Briefly, we summed the mutation rate (mu_agg) for each substitution, context, and methylation level across the exome. We then applied the appropriate plateau model (autosomal/pseudoautosomal, chromosome X, chromosome Y) and adjusted CpG vs. non-CpG sites separately. After applying the appropriate plateau model, we applied the coverage model to low coverage (median coverage < 40x) sites to create the final adjusted mutation rate (mu_adj). We then aggregated the raw mutation rate sum (mu_agg) and mutation rate (mu_adj) per transcript to get the total mutation rate sum and proportion of expected missense variation per transcript.

We implemented a minimum number of expected missense variants to prevent finding breakpoint positions that would create very small (i.e., a handful of base pairs in size) transcript subsections (see Supplementary Note).

We applied a likelihood ratio test to determine whether the missense observed/expected (OE) ratio was uniform along a transcript or whether a transcript had evidence of distinct sections of missense constraint. We used the observed and expected missense counts to search for positions that would divide a transcript into two or more regions with varying levels of missense depletion. For our analyses, we assume that the observed missense counts should follow a Poisson distribution around the expected missense counts. We defined our null model as transcripts not having any evidence of regional variability in missense depletion (where the expectation, the OE ratio, is consistent across the length of the transcript). Our alternative model was that transcripts exhibited evidence of distinct sections of missense depletion (OE ratio calculated per transcript subsection). Because the alternative model should always have a better fit than the null model, we require a chi square value above a given threshold (p = 0.001) to establish significance. We used the following formulas to determine the significance of a breakpoint that would split a transcript into two sections, A and B:

-
$p_{0}=Pois(obs_{A},exp_{A}*OE)*Pois(obs_{B},exp_{B}*OE)$

-
$p_{1}=Pois(obs_{A},exp_{A}*OE_{A})*Pois(obs_{B},exp_{B}*OE_{B})$

-
$\chi^{2}=2(ln(p_{1})-ln(p_{0}))$

where OE is the missense observed/expected ratio across the entire transcript, obs_A_ is the number of observed missense variants in transcript section A, exp_A_ is the number of expected missense variants in transcript section A, OE_A_ is the OE ratio across transcript section A, obs_B_ **is** the number of observed missense variants in transcript section B, exp_B_ is the number of expected missense variants in transcript section A, OE_B_ is the OE ratio across transcript section B, and Pois is the Poisson likelihood.

We used the following formulas to determine the significance of a breakpoint that would split a transcript into three sections, A, B, and C:

-
$p_{0}=Pois(obs_{A},exp_{A}*OE)*Pois(obs_{B},exp_{B}*OE)*Pois(obs_{C},exp_{C}*OE)$

-
$p_{1}=Pois(obs_{A},exp_{A}*OE_{A})*Pois(obs_{B},exp_{B}*OE_{B})*Pois(obs_{C},exp_{C}*OE_{C})$

-
$\chi^{2}=2(ln(p_{1})-ln(p_{0}))$

where OE is the missense observed/expected ratio across the entire transcript, obs_A_ is the number of observed missense variants in transcript section A, exp_A_ is the number of expected missense variants in transcript section A, OE_A_ is the OE ratio across transcript section A, obs_B_ is the number of observed missense variants in transcript section B, exp_B_ is the number of expected missense variants in transcript section A, OE_B_ is the OE ratio across transcript section B, obs_C_ is the number of observed missense variants in transcript section C, exp_C_ is the number of expected missense variants in section C, and Pois is the Poisson likelihood.

For the purposes of our analyses, all transcript subsections with more observed variants than expected were capped at an OE of 1, as we were looking for areas of missense depletion and not missense enrichment. We also converted the expected counts for transcript subsections with zero expected variants from 0 to 10^−9^ to prevent nonfinite OE values.

To search for a single breakpoint that would divide a transcript into two subsections, we calculated chi square statistics (as discussed above) to conduct likelihood ratio tests simultaneously for every eligible position within a transcript. The positions we considered were positions with a possible missense variant substitution that had at least 16 expected missense counts in either direction (i.e., both transcript subsections created by dividing the transcript at this point would have at least 16 expected missense variants). We then aggregated chi square values across each transcript to find the maximum value per transcript, and we marked any positions as breakpoints if the chi square calculated at that position was equal to the maximum chi square value over all sites in the transcript and significant at p = 0.001.

Any transcripts that did not have a single significant breakpoint moved forwards into our two simultaneous breaks search flow. In this search flow, we again calculated chi square statistics to conduct likelihood ratio tests for every eligible position pair. For every position with a possible missense, we calculated the chi square statistic of that position paired with each possible position downstream as long as the two positions created transcript subsections with at least 16 expected missense variants (i.e., all three of the transcript subsections created would have at least 16 expected missense variants). Because of the large number of pairwise computations, this step is the most computationally intensive portion of our algorithm. After completing the single and two simultaneous break search workflows, we merged the results from both search types.

Our breakpoint search flow is recursive, and the steps are as follows: Search for a single significant breakpoint dividing a transcript into two subsections. If no single significant breakpoint was found in the transcript, search for two simultaneous breakpoints. Merge the results from the single and two simultaneous breakpoint searches. Repeat the steps above, treating each separate transcript subsection as if it were an independent transcript, until no more significant breakpoints are found.

### Modeling deleteriousness of missense substitution classes with missense constraint

We incorporated two MCR OE-based metrics to measure the increased deleteriousness of amino acid substitution classes (e.g., Met to Tyr) in functionally important areas of proteins: the overall OE for each substitution and the second derivative of this OE value per OE bin of missense constraint (Supplementary Fig. 14). To calculate the first metric, the substitution overall OE, we divided the total number of rare, high quality variants (see gnomAD variants) causing that substitution by the total number of expected variants (see Modeling of mutation rates and expected neutral missense variation). To calculate the second metric, the substitution OE second derivative, we aggregated the OEs of each substitution by MCR OE bin in 10 bins from 0 to 1.0+ (i.e., for the 0-0.1 OE bin, we calculated all of the observed substitutions that occurred within regions with a OE between 0 and 0.1 and divided that number by the total number of expected substitutions occurring in those regions).

### Modeling deleteriousness of individual missense variants

We designed a missense variant deleteriousness predictor to explicitly incorporate information on amino acid substitution class and position-specific variant effects. A logistic regression model was first trained to differentiate pathogenic from benign missense variants. The pathogenic training set consisted of high-quality ClinVar variants (see ClinVar variants) labeled as pathogenic or likely pathogenic in 2,987 likely-haploinsufficient genes, defined as having probability of haploinsufficiency (pHaplo) ≥ 0.86^41^, or in 366 genes with DD associations in G2P through non-LoF mechanisms. The latter gene set was created by filtering on the G2P DD panel (accessed October 6, 2023) to select genes where: 1. confidence_category is either definitive or strong evidence, 2. allelic_requirement was monoallelic, and 3. mutation_consequence included altered gene product structure or increased gene product level. The benign training set consisted of high-quality common variants as described in gnomAD variants. Variants matching criteria for both the benign and pathogenic sets were removed from the training data. We evaluated models with all possible combinations of the following complementary features: amino acid substitution overall OE and OE second derivative (see Modeling deleteriousness of missense substitution classes); BLOSUM^38^ and Grantham^39^ scores of amino acid substitution class severity; the local missense constraint level of a variant (missense OE across the MCR if applicable, else across the transcript); and PolyPhen-2^40^. We selected BLOSUM, Grantham, and PolyPhen-2 because of the orthogonal information added on top of our OE-based (and therefore population allele frequency-dependent) metrics. For each model, only variants with all relevant annotations were used in training the regression model and the subsequent calculations to produce deleteriousness scores. The deleteriousness score prediction for any missense variant i is given as:

$$d_{i}=-log_{10}(m_{i}∕M)\;\;m_{i}=max(0.83,f_{i})$$

where $d_{i}$ is the deleteriousness score prediction, $f_{i}$ is the number of common missense variants with a fitted value from the regression that is less than the fitted value for variant i, and M is the number of common missense variants (equivalent to the number of benign training variants for the regression). $m_{i}$ is set to have a minimum value of 0.83 to avoid a mathematical error in the log when the fitted value for a given variant is less than those of all common variants. Larger values of $d_{i}$ indicate stronger predicted-deleteriousness. The best model was chosen to be the model featurized with all six possible features. The training set for this model consisted of 64,023 benign variants and 12,955 pathogenic variants. This model was then applied to produce MPC scores for the 68,576,965 possible exome-wide missense variants with all features, and the distribution of these MPC scores is given in Supplementary Figs. 10, 11, and 12.

### Comparison of MPC to other predictors

We compared our model to the following missense deleteriousness predictors: AlphaMissense^42^, Constrained Coding Regions (CCRs)^9^, MVP^46^, M-CAP^43^, PrimateAI-3D^45^, REVEL^44^, CADD^47,48^, PolyPhen-2^40^, and SIFT^49^. We annotated the case and control *de novo* missense variants described in Rare and de novo variants from developmental cohorts and ranked the variants based on their annotated scores. To assess each predictor's ability to stratify case and control variation, we assessed the proportion of case to control variants among the variants with the top 10% for each score and compared this number to the overall proportion of case to control variation using a Fisher exact test.

## Supplementary Material

Supplement 1

Supplement 2

Supplement 3

## Data Availability

The missense constraint regions (MCRs) are displayed on the gnomAD v2 browser (https://gnomad.broadinstitute.org) and available for download on the gnomAD website (https://gnomad.broadinstitute.org/downloads#v2) and in the gnomAD v2 public datasets on Google, Amazon, and Microsoft clouds. MPC scores for all possible variants in canonical transcripts is available in the gnomAD v2 public datasets on Google (gs://gcp-public-data--gnomad/release/2.1.1/regional_missense_constraint/gnomad_v2.1.1_mpc.ht). gnomAD v2 exome, genome, and coverage data and the table of all possible single nucleotide polymorphisms used to calculate mutational models and search for MCRs are also available in the gnomAD public buckets and are easily accessed using code in the gnomAD Hail utilities GitHub repository (https://github.com/broadinstitute/gnomad_methods/blob/7c0c994883f321492a48962674d5caeb289df4c7/gnomad/resources/grch37/gnomad.py#L107 and https://github.com/broadinstitute/gnomad_methods/blob/7c0c994883f321492a48962674d5caeb289df4c7/gnomad/utils/vep.py#L161). ClinVar data were downloaded from ClinVar's FTP server (https://ftp.ncbi.nlm.nih.gov/pub/clinvar/). *De novo* variants were extracted from the supplemental files of the cited studies. AlphaMissense scores were downloaded from https://github.com/google-deepmind/alphamissense. CCRs were downloaded from https://github.com/quinlan-lab/ccrhtml. MCAP scores were downloaded from http://bejerano.stanford.edu/MCAP/. REVEL scores were downloaded from https://sites.google.com/site/revelgenomics/. PrimateAI-3D scores were downloaded from https://primad.basespace.illumina.com/download. MVP scores were downloaded from https://figshare.com/articles/dataset/Predicting_pathogenicity_of_missense_variants_by_deep_l_earning/13204118. PolyPhen-2 and SIFT scores were obtained from VEP^56^. CADD scores were downloaded from the CADD website (https://cadd.gs.washington.edu/download). phyloP scores were downloaded from the UCSC browser (https://genome.ucsc.edu/cgi-bin/hgTrackUi?db=hg38&g=cons241way).

## Code availability

Code to determine missense constraint regions (MCRs) and calculate MPC is available at https://github.com/broadinstitute/regional_missense_constraint. Code used to generate the mutational models is available at https://github.com/broadinstitute/gnomad_lof and https://github.com/broadinstitute/gnomad-constraint. The Hail library is available at https://hail.is/.

## References

[1] Samocha K. E. Regional missense constraint improves variant deleteriousness prediction. bioRxiv 148353 (2017) doi:10.1101/148353.

[2] Gussow A. B., Petrovski S., Wang Q., Allen A. S. & Goldstein D. B. The intolerance to functional genetic variation of protein domains predicts the localization of pathogenic mutations within genes. Genome Biol. 17, 9 (2016). 26781712 10.1186/s13059-016-0869-4 PMC4717634

[3] Wiel L., Venselaar H., Veltman J. A., Vriend G. & Gilissen C. Aggregation of population-based genetic variation over protein domain homologues and its potential use in genetic diagnostics. Hum. Mutat. 38, 1454–1463 (2017). 28815929 10.1002/humu.23313 PMC5656839

[4] Sivley R. M., Dou X., Meiler J., Bush W. S. & Capra J. A. Comprehensive Analysis of Constraint on the Spatial Distribution of Missense Variants in Human Protein Structures. Am. J. Hum. Genet. 102, 415–426 (2018). 29455857 10.1016/j.ajhg.2018.01.017 PMC5985282

[5] Lal D. Gene family information facilitates variant interpretation and identification of disease-associated genes in neurodevelopmental disorders. Genome Med. 12, 28 (2020). 32183904 10.1186/s13073-020-00725-6 PMC7079346

[6] Zhang X. Genetic constraint at single amino acid resolution improves missense variant prioritisation and gene discovery. (2022) doi:10.1101/2022.02.16.22271023. PMC11238507 38992748

[7] Traynelis J. Optimizing genomic medicine in epilepsy through a gene-customized approach to missense variant interpretation. Genome Res. 27, 1715–1729 (2017). 28864458 10.1101/gr.226589.117 PMC5630035

[8] Perszyk R. E., Kristensen A. S., Lyuboslavsky P. & Traynelis S. F. Three-dimensional missense tolerance ratio analysis. Genome Res. 31, 1447–1461 (2021). 34301626 10.1101/gr.275528.121 PMC8327912

[9] Havrilla J. M., Pedersen B. S., Layer R. M. & Quinlan A. R. A map of constrained coding regions in the human genome. Nat. Genet. 51, 88–95 (2019). 30531870 10.1038/s41588-018-0294-6 PMC6589356

[10] Silk M. MTR3D: identifying regions within protein tertiary structures under purifying selection. Nucleic Acids Res. 49, W438–W445 (2021). 34050760 10.1093/nar/gkab428 PMC8265191

[11] Hicks M., Bartha I., di Iulio J., Venter J. C. & Telenti A. Functional characterization of 3D protein structures informed by human genetic diversity. Proc. Natl. Acad. Sci. U. S. A. 116, 8960–8965 (2019). 30988206 10.1073/pnas.1820813116 PMC6500140

[12] Li B., Roden D. M. & Capra J. A. The 3D mutational constraint on amino acid sites in the human proteome. Nat. Commun. 13, 3273 (2022). 35672414 10.1038/s41467-022-30936-x PMC9174330

[13] Karczewski K. J. The mutational constraint spectrum quantified from variation in 141,456 humans. Nature 581, 434–443 (2020). 32461654 10.1038/s41586-020-2308-7 PMC7334197

[14] Landrum M. J. ClinVar: improvements to accessing data. Nucleic Acids Res. 48, (2020). 10.1093/nar/gkz972 PMC6943040 31777943

[15] Kaplanis J. Evidence for 28 genetic disorders discovered by combining healthcare and research data. Nature 586, 757–762 (2020). 33057194 10.1038/s41586-020-2832-5 PMC7116826

[16] Fu J. M. Rare coding variation provides insight into the genetic architecture and phenotypic context of autism. Nat. Genet. 54, 1320–1331 (2022). 35982160 10.1038/s41588-022-01104-0 PMC9653013

[17] 1000 Genomes Project Consortium A global reference for human genetic variation. Nature 526, 68–74 (2015). 26432245 10.1038/nature15393 PMC4750478

[18] Lek M. Analysis of protein-coding genetic variation in 60,706 humans. Nature 536, 285–291 (2016). 27535533 10.1038/nature19057 PMC5018207

[19] Taliun D. Sequencing of 53,831 diverse genomes from the NHLBI TOPMed Program. Nature 590, 290–299 (2021). 33568819 10.1038/s41586-021-03205-y PMC7875770

[20] Backman J. D. Exome sequencing and analysis of 454,787 UK Biobank participants. Nature 599, 628–634 (2021). 34662886 10.1038/s41586-021-04103-z PMC8596853

[21] All of Us Research Program Genomics Investigators. Genomic data in the All of Us Research Program. Nature (2024) doi:10.1038/s41586-023-06957-x. PMC10937371 38374255

[22] Petrovski S. The Intolerance of Regulatory Sequence to Genetic Variation Predicts Gene Dosage Sensitivity. PLoS Genet. 11, e1005492 (2015). 26332131 10.1371/journal.pgen.1005492 PMC4557908

[23] Samocha K. E. A framework for the interpretation of de novo mutation in human disease. Nat. Genet. 46, 944–950 (2014). 25086666 10.1038/ng.3050 PMC4222185

[24] Weghorn D. Applicability of the Mutation-Selection Balance Model to Population Genetics of Heterozygous Protein-Truncating Variants in Humans. Mol. Biol. Evol. 36, 1701–1710 (2019). 31004148 10.1093/molbev/msz092 PMC6738481

[25] Agarwal I., Fuller Z. L., Myers S. R. & Przeworski M. Relating pathogenic loss-of-function mutations in humans to their evolutionary fitness costs. Elife 12, (2023). 10.7554/eLife.83172 PMC9937649 36648429

[26] Kosmicki J. A. Refining the role of de novo protein-truncating variants in neurodevelopmental disorders by using population reference samples. Nat. Genet. 49, 504–510 (2017). 28191890 10.1038/ng.3789 PMC5496244

[27] Bamshad M. J., Nickerson D. A. & Chong J. X. Mendelian Gene Discovery: Fast and Furious with No End in Sight. Am. J. Hum. Genet. 105, 448–455 (2019). 31491408 10.1016/j.ajhg.2019.07.011 PMC6731362

[28] Seaby E. G., Rehm H. L. & O’Donnell-Luria A. Strategies to Uplift Novel Mendelian Gene Discovery for Improved Clinical Outcomes. Front. Genet. 12, 674295 (2021). 34220947 10.3389/fgene.2021.674295 PMC8248347

[29] Kapplinger J. D. Enhancing the Predictive Power of Mutations in the C-Terminus of the KCNQ1-Encoded Kv7.1 Voltage-Gated Potassium Channel. J. Cardiovasc. Transl. Res. 8, 187–197 (2015). 25854863 10.1007/s12265-015-9622-8 PMC4907365

[30] Richards S. Standards and guidelines for the interpretation of sequence variants: a joint consensus recommendation of the American College of Medical Genetics and Genomics and the Association for Molecular Pathology. Genet. Med. 17, 405–424 (2015). 25741868 10.1038/gim.2015.30 PMC4544753

[31] Whiffin N. CardioClassifier: disease- and gene-specific computational decision support for clinical genome interpretation. Genet. Med. 20, 1246–1254 (2018). 29369293 10.1038/gim.2017.258 PMC6558251

[32] Kury S. Rare germline heterozygous missense variants in BRCA1-associated protein 1, BAP1, cause a syndromic neurodevelopmental disorder. Am. J. Hum. Genet. 109, 361–372 (2022). 35051358 10.1016/j.ajhg.2021.12.011 PMC8874225

[33] Hamosh A., Amberger J. S., Bocchini C., Scott A. F. & Rasmussen S. A. Online Mendelian Inheritance in Man (OMIM^®^): Victor McKusick’s magnum opus. Am. J. Med. Genet. A 185, 3259–3265 (2021). 34169650 10.1002/ajmg.a.62407 PMC8596664

[34] Thormann A. Flexible and scalable diagnostic filtering of genomic variants using G2P with Ensembl VEP. Nat. Commun. 10, 2373 (2019). 31147538 10.1038/s41467-019-10016-3 PMC6542828

[35] Christmas M. J. Evolutionary constraint and innovation across hundreds of placental mammals. Science 380, eabn3943 (2023). 37104599 10.1126/science.abn3943 PMC10250106

[36] Landrum M. J. ClinVar: improving access to variant interpretations and supporting evidence. Nucleic Acids Res. 46, D1062–D1067 (2018). 29165669 10.1093/nar/gkx1153 PMC5753237

[37] Pejaver V. Calibration of computational tools for missense variant pathogenicity classification and ClinGen recommendations for PP3/BP4 criteria. Am. J. Hum. Genet. 109, 2163–2177 (2022). 36413997 10.1016/j.ajhg.2022.10.013 PMC9748256

[38] Henikoff S. & Henikoff J. G. Amino acid substitution matrices from protein blocks. Proc. Natl. Acad. Sci. U. S. A. 89, 10915–10919 (1992). 1438297 10.1073/pnas.89.22.10915 PMC50453

[39] Grantham R. Amino acid difference formula to help explain protein evolution. Science 185, 862–864 (1974). 4843792 10.1126/science.185.4154.862

[40] Adzhubei I. A. A method and server for predicting damaging missense mutations. Nat. Methods 7, 248–249 (2010). 20354512 10.1038/nmeth0410-248 PMC2855889

[41] Collins R. L. A cross-disorder dosage sensitivity map of the human genome. Cell 185, 3041–3055.e25 (2022). 35917817 10.1016/j.cell.2022.06.036 PMC9742861

[42] Cheng J. Accurate proteome-wide missense variant effect prediction with AlphaMissense. Science 381, eadg7492 (2023). 37733863 10.1126/science.adg7492

[43] Jagadeesh K. A. M-CAP eliminates a majority of variants of uncertain significance in clinical exomes at high sensitivity. Nat. Genet. 48, 1581–1586 (2016). 27776117 10.1038/ng.3703

[44] Ioannidis N. M. REVEL: An Ensemble Method for Predicting the Pathogenicity of Rare Missense Variants. Am. J. Hum. Genet. 99, 877–885 (2016). 27666373 10.1016/j.ajhg.2016.08.016 PMC5065685

[45] Gao H. The landscape of tolerated genetic variation in humans and primates. Science 380, eabn8153 (2023). 10.1126/science.abn8197 PMC10713091 37262156

[46] Qi H. MVP predicts the pathogenicity of missense variants by deep learning. Nat. Commun. 12, 510 (2021). 33479230 10.1038/s41467-020-20847-0 PMC7820281

[47] Kircher M. A general framework for estimating the relative pathogenicity of human genetic variants. Nat. Genet. 46, 310–315 (2014). 24487276 10.1038/ng.2892 PMC3992975

[48] Rentzsch P., Schubach M., Shendure J. & Kircher M. CADD-Splice-improving genome-wide variant effect prediction using deep learning-derived splice scores. Genome Med. 13, 31 (2021). 33618777 10.1186/s13073-021-00835-9 PMC7901104

[49] Ng P. C. & Henikoff S. SIFT: Predicting amino acid changes that affect protein function. Nucleic Acids Res. 31, 3812–3814 (2003). 12824425 10.1093/nar/gkg509 PMC168916

[50] Fuller Z. L., Berg J. J., Mostafavi H., Sella G. & Przeworski M. Measuring intolerance to mutation in human genetics. Nat. Genet. 51, 772–776 (2019). 30962618 10.1038/s41588-019-0383-1 PMC6615471

[51] Jin S. C. Contribution of rare inherited and de novo variants in 2,871 congenital heart disease probands. Nat. Genet. 49, 1593–1601 (2017). 28991257 10.1038/ng.3970 PMC5675000

[52] Bishop M. R. Genome-wide Enrichment of De Novo Coding Mutations in Orofacial Cleft Trios. Am. J. Hum. Genet. 107, 124–136 (2020). 32574564 10.1016/j.ajhg.2020.05.018 PMC7332647

[53] Qiao L. Rare and de novo variants in 827 congenital diaphragmatic hernia probands implicate LONP1 as candidate risk gene. Am. J. Hum. Genet. 108, 1964–1980 (2021). 34547244 10.1016/j.ajhg.2021.08.011 PMC8546037

[54] EuroEPINOMICS-RES Consortium, Epilepsy Phenome/Genome Project & Epi4K Consortium. De novo mutations in synaptic transmission genes including DNM1 cause epileptic encephalopathies. Am. J. Hum. Genet. 95, 360–370 (2014). 25262651 10.1016/j.ajhg.2014.08.013 PMC4185114

[55] Roadmap Epigenomics Consortium Integrative analysis of 111 reference human epigenomes. Nature 518, 317–330 (2015). 25693563 10.1038/nature14248 PMC4530010

[56] McLaren W. The Ensembl Variant Effect Predictor. Genome Biol. 17, 122 (2016). 27268795 10.1186/s13059-016-0974-4 PMC4893825

