# Supplementary material for: The landscape of regional missense mutational intolerance quantified from 125,748 exomes
Source: bioRxiv. 2024 Apr 13:2024.04.11.588920. Preprint. [Version 1] doi: 10.1101/2024.04.11.588920 (PMC11030311; doi:10.1101/2024.04.11.588920)
Supplement: Supplement 1 [file media-1.docx]

## Supplementary Text

### Missense constraint region size

Transcripts where we did not find evidence of regional variability in missense constraint are deemed to have one missense constraint region (MCR) from the start to the end of the CDS. In transcripts exhibiting evidence of regional missense constraint, the minimum MCR coding length is 49bp, and the median is 461 bp (**Supplementary Fig. 1a**). In these transcripts, we observe that regions under stronger constraint tend to be smaller: MCRs with OE < 0.4 have a median coding length of 197bp, while MCRs with OE ≥ 0.4 have a median coding length of 624bp (Wilcoxon p < 10^-50^; **Supplementary Fig. 1b, 1c**).

#### Determining minimum section size

We ran a power analysis to decide the minimum MCR size that our search should emit. We plotted the simplest single break (single breakpoint dividing transcript into two MCRs) search scenario. We modeled the case where one MCR had a missense observed/expected ratio (OE) of 0, and the other MCR had an OE of 1, as this is the maximum difference in OE values. We determined that the p-value for exome-wide significance was 2.7x10^-6^ (0.05 / 18629 transcripts), and we used the power plot to determine that at p = 2.7x10^-6^, the minimum number of expected variants on either side of the breakpoint position was 16.

### Technical factors influencing quantification of missense depletion

#### Coverage

We binned MCRs into four bins based on their median coverage: 1-10x, 10-20x, 20-30x, and > 30x. A larger proportion of MCRs are highly missense-depleted (MCR missense OE < 0.4) in the lowest coverage bin (169/461; 1-10x) as compared to the highest coverage bin (4435/16007; > 30x; Fisher exact p = 0.0028). We caution against overinterpretation of highly constrained MCRs within low coverage sequences, though this is a small (<1%) portion of our overall dataset.

#### Proportion expression across transcripts

We assessed whether MCRs that are unconstrained (MCR missense OE > 0.9 based on missense OE threshold with supporting evidence for BP4; see Supplementary Table 2) are significantly enriched for having low RNA expression output, quantified using the pext metric[^1^](https://paperpile.com/c/UaG5kk/LKMr). For transcripts with at least two MCRs, we calculated median pext scores across each MCR and assessed MCRs in two categories: MCRs with low median pext (≤ 0.1) and MCRs with high median pext (≥ 0.9). More MCRs with low median pext scores (42.4%; 218/514) are unconstrained as compared to MCRs with high median pext scores (pext ≥ 0.9; 2055/7319; Fisher exact p = 2.1x10^-6^), indicating these regions may be included in the Ensembl canonical transcript that we analyze in our models but may not be the most biologically appropriate gene model.

### Justification of missense OE thresholds in enrichment tests

To determine the missense OE threshold used to define missense constrained vs. unconstrained regions, we compared the rate of *de novo* missense variation in individuals with developmental disorders (DD; n = 31,058)[^2^](https://paperpile.com/c/UaG5kk/86n1) to the rate of *de novo* missense variation in sibling controls (n = 5,492)[^3^](https://paperpile.com/c/UaG5kk/jRYNZ) across five different bins of missense OE (**Supplementary Fig. 7**). Given that only a subset of the *de novo* missense variants in DD individuals are causal for disease, we expected that regions intolerant of missense variation would have higher rate ratios (RR), and regions tolerant of missense variation would have a RR close to 1 (equal rates of *de novo* missense variation in cases and controls reflecting background mutation rates). We observed that the bins with missense OE < 0.4 had significantly higher RRs than the bins with missense OE > 0.4, with a particularly strong enrichment (RR = 6.6) of *de novo* missense variants from cases vs. controls in the first missense OE bin (OE < 0.2).

In addition, after applying previously established probabilistic frameworks[^4^](https://paperpile.com/c/UaG5kk/4VI4), we found that a missense OE ≤ 0.37 met supporting and missense OE ≤ 0.21 met moderate level evidence for pathogenicity (**Supplementary Table 2**). This calibration corroborated our selection of missense OE < 0.4 as the cutoff between missense constrained and unconstrained regions and the usage of missense OE < 0.2 to assess enrichment of *de novo* missense variants in individuals with DD vs. in autistic individuals.

### Comparing missense constraint to pLoF constraint

We found that 6% (354) of 5,605 LoF-constrained genes (within the first three LOEUF deciles) are missense-constrained (missense OE < 0.4) across the entire transcript. An additional 41% (2,305) genes are missense-constrained at the same threshold when assessing their minimum MCR OE. In the last three deciles of LOEUF, only 0.7% (36/5258) genes are missense-constrained at the transcript level, and only an additional 3% (118) genes are MCR missense-constrained.

### Comparing missense constraint to OMIM disease association

64% (1697/2659) of genes that are LOEUF- and MCR missense-constrained (first three deciles of LOEUF and MCR with OE < 0.4) do not have disease associations in OMIM[^5^](https://paperpile.com/c/UaG5kk/disZ). Genes not associated with disease in OMIM tend to have higher minimum MCR missense OEs (i.e., less constrained) than genes with dominant disease association (Wilcoxon p < 10^-50^) but lower (i.e., more constrained) than genes with recessive disease association (Wilcoxon p = 5.9x10^-8^), suggesting substantial heterozygous selection in a subset of as-of-yet disease-unassociated genes (**Supplementary Fig. 3**).

### Comparing missense constraint to conservation

We investigated potential sites of divergence between recent selection on human variation (measured by missense constraint) compared to selection over longer timescales (measured by evolutionary conservation in placental mammals, phyloP[^6^](https://paperpile.com/c/UaG5kk/1CJ4I)). Genes with more conserved coding sequences also tended to be more overall depleted of human missense variation (Spearman ρ = 0.56, p < 10^-50^), consistent with previous correlations found between stronger mammalian conservation and lower human allele frequency[^6^](https://paperpile.com/c/UaG5kk/1CJ4I) (**Supplementary Fig. 6a, c**). However, when moving to our subgenic human constraint metric, we discover a substantial number of MCRs that appear strongly constrained against missense variants in humans but widely unconserved across mammals (Spearman ρ = 0.54, p < 10^-50^), potentially pointing to human-specific negative selection pressures that are obscured when smoothing constraint over whole transcripts (**Supplementary Fig. 6b, d**). There is no significant difference in location of genes with these MCRs on autosomes vs. allosomes (Fisher exact p = 0.2). We do not see a similar accumulation of mammalian-conserved yet human-unconstrained regions, indicating that negative selection pressures that have been maintained across mammalian evolution are unlikely to be waived in humans.

We found 102 autosomal genes have at least one constrained MCR (missense OE ≤ 0.4) where ≥ 70% of coding bases are unconserved across placental mammals (phyloP < 2.7[^6^](https://paperpile.com/c/UaG5kk/1CJ4I)). A Gene Ontology (GO) enrichment analysis of these genes against a background set of all 4,896 autosomal genes with two or more MCRs using shinyGO v0.80[^7^](https://paperpile.com/c/UaG5kk/170e) revealed no significant enrichment among biological processes, cellular components, or molecular function. Searching against OMIM diseases revealed a 13-fold enrichment for deafness, with five hit genes out of 57 total in the pathway with two or more MCRs (FDR p = 1.2x10^-4^).

### Partitioning heritability enrichment over missense constraint

Heritability enrichment was computed as in Finucane *et al*.[^8^](https://paperpile.com/c/UaG5kk/Dw1Y) Briefly, for each trait, per-SNP heritability was calculated using LD score regression, and heritability enrichment was calculated for each category of SNPs as the proportion of heritability ascribed to SNPs in the category divided by the proportion of SNPs in that category. This study partitioned heritability enrichment over five SNP categories, comprising coding SNPs in each missense constraint quintile. Quintiles were computed over MCRs in all 18,629 transcripts. The boundaries on missense OE corresponding to these five categories were: 0.0-0.4622, 0.4622-0.7543, 0.7543-0.8994, 0.8994-1.0160, and 1.0160+. Heritability enrichment was computed across traits analyzed in Karczewski *et al*.[^9^](https://paperpile.com/c/UaG5kk/h5rG), removing traits with a maximum pairwise correlation greater than 0.2 for a total of 268 independent traits analyzed in this study. The mean heritability enrichment for all coding SNPs in MCRs was 3.03 with a standard deviation of 9.78.

### Clinical calibration of regional constraint metrics

**Datasets.** We filtered to retain genes that had at least one pathogenic missense variant in ClinVar (Accessed December 7, 2023) and then selected missense variants in those genes that had at least a one-star review status and one of the following classifications: pathogenic, pathogenic/likely pathogenic, likely pathogenic, benign, benign/likely benign, or likely benign. Variants with an allele frequency (AF) ≥ 1% in gnomAD v2.1.1 or with conflicting classifications were removed. This process yielded a total of 87,814 ClinVar variants from 3,173 genes. We also retrieved missense variants from gnomAD v2.1.1 exomes and genomes in the genes where we collected ClinVar variants as described above. We retained only QC-pass variants ("PASS" in the "FILTER" column") and removed variants with an AF ≥ 1%. This resulted in a set of 1,108,335 gnomAD variants from 2,933 genes.

**Calibration.** We calibrated MCR missense OE exome-wide as well as specifically in transcripts with multiple MCRs, along with two other metrics of regional constraint, COSMIS[^10^](https://paperpile.com/c/UaG5kk/5SNQ) and CCRs[^11^](https://paperpile.com/c/UaG5kk/auC5), to evaluate their utility in clinical variant classification. We first annotated the ClinVar and gnomAD variants from the datasets described above with scores from each metric. Using the method developed by Pejaver *et al.*[*^4^*](https://paperpile.com/c/UaG5kk/4VI4) and the prior probability of pathogenicity of 4.41% established therein, we computed the local posterior probability curves for all score values given by each method. ClinVar variants were used with their classification labels to estimate the local values of the posterior probability of pathogenicity, and gnomAD variants were used to smooth out the posterior estimates (≥1% gnomAD variants required in each local window). Consequently, we defined score threshold ranges for each strength of evidence (supporting, moderate, strong, and very strong) for pathogenicity and benignity based on the ACMG/AMP criteria. For each metric, we plotted the local posterior probability curve estimate along with the one-sided 95% confidence interval, calculated on the more stringent side, determined using 10,000 bootstrapping iterations (**Fig. 4b** and **Supplementary Fig. 9**). The final thresholds for each tool as determined with bootstrapping are provided in **Supplementary Table 2**.

### Association testing in neurodevelopmental disorders (NDDs) with MPC

To further pinpoint enrichment across specific regimes within the spectrum of predicted-deleteriousness, we compared case and control missense rates stratified by MPC bins. We additionally stratified by localization of variants to known NDD-associated genes[^3^](https://paperpile.com/c/UaG5kk/jRYNZ) in order to probe the genetic architecture of NDDs that remains to be mapped. We created three bins of MPC scores (<1.6, 1.6-2.6, ≥2.6) defined by the MPC thresholds at which the NDD case-control *de novo* rate ratio of missense variants met the rate ratio of protein-truncating variants in LoF-constrained genes; MPC ≥2.6 corresponds to enrichment in the first two LOEUF deciles, and MPC between 1.6 and 2.6 to enrichment in the third and fourth LOEUF deciles.

The confidence interval of the relative difference statistics is calculated based on a binomial test, with probability of the binomial distribution equal to (a) for *de novo*, number of affected offspring divided by the total number of offspring; (b) for inherited, 0.5; and (c) for case/control, the number of cases divided by the total number of samples in the case/control data.

### MPC evaluation against other missense deleteriousness prediction metrics

Every metric analyzed except SIFT consistently predicts *de novo* variants to have equal or stronger deleteriousness on average in cases compared to controls, indicating simply using protein sequence homology is likely insufficient for predicting comparative disease effects among human *de novo* missense variants.

## Supplementary Figures


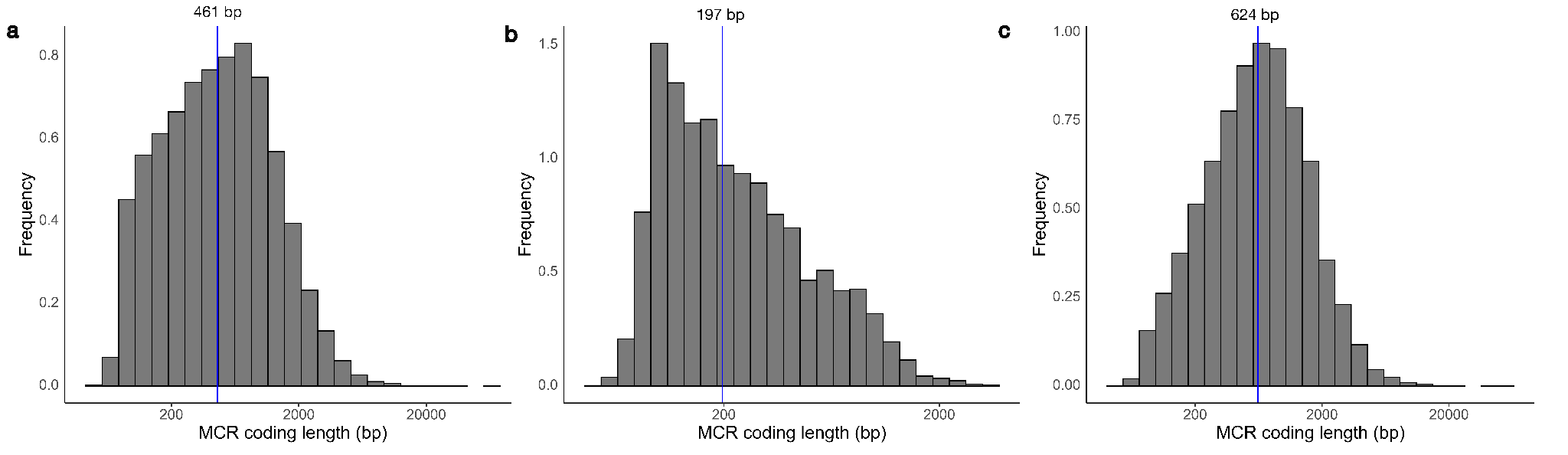


**Supplementary Fig. 1**: Missense constraint region (MCR) coding length across 5,127 transcripts harboring regional variability in missense constraint.

**a**, Distribution of coding length. Median is 461 base pairs. **b**, Distribution of coding length for MCRs with strong missense constraint (missense OE < 0.4). Median is 197 base pairs. **c,** Distribution of coding length for MCRs with weaker missense constraint (missense OE ≥ 0.4). Median is 624 base pairs.

**
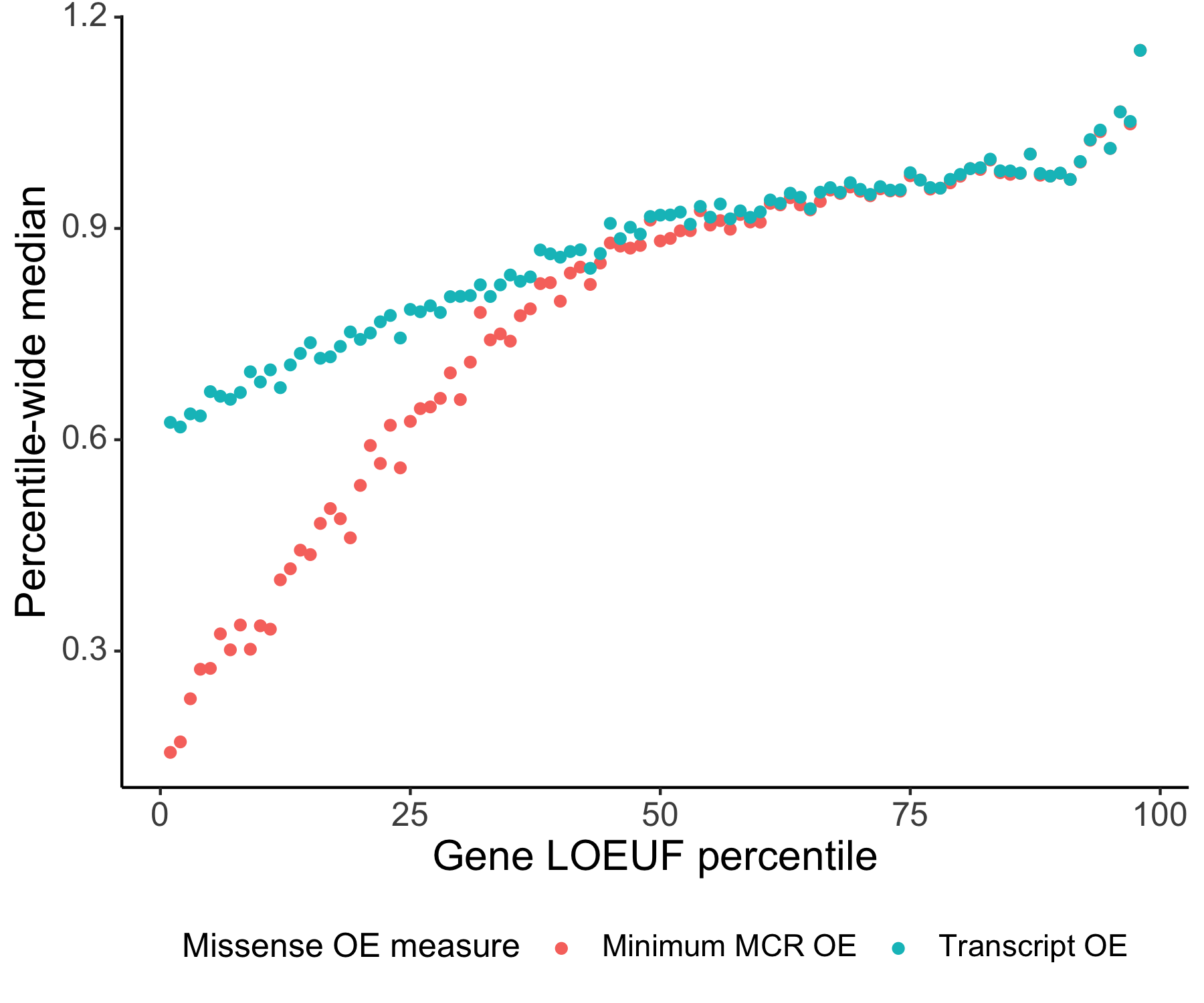
**

**Supplementary Fig. 2**: Loss-of-function (LoF)-intolerant transcripts tend to be intolerant to missense variation.

Red: Minimum missense constraint region (MCR)-level missense OE; blue: transcript-level missense OE. The medians over transcripts within each LOEUF percentile are shown for each missense OE measure.


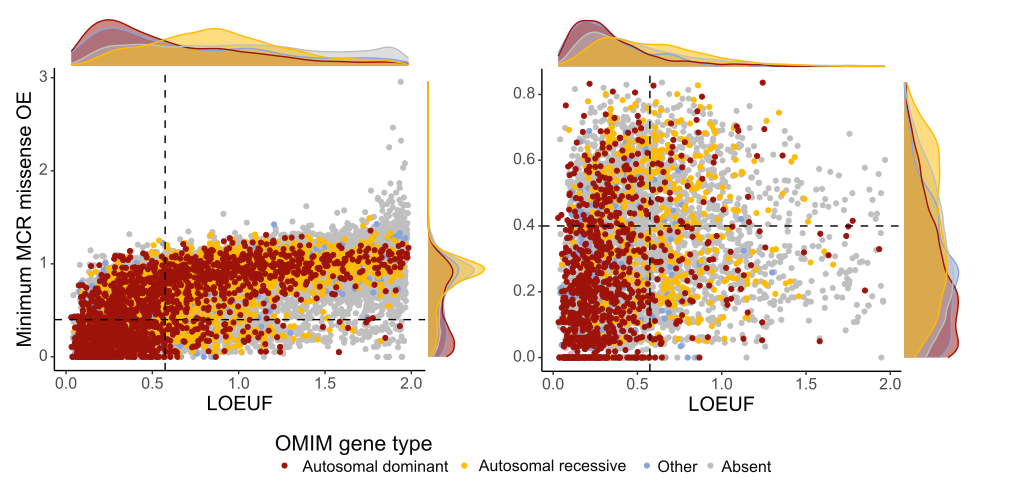


**Supplementary Fig. 3**: Loss-of-function (LoF) and missense constraint suggest additional potential for disease gene discovery. Left: all 18,629 genes; right: 5,127 genes with two or more missense constraint regions (MCRs). Black dashed lines at 30th percentile of LOEUF and minimum MCR missense OE of 0.4 demarcate LoF- and missense-constrained transcripts.


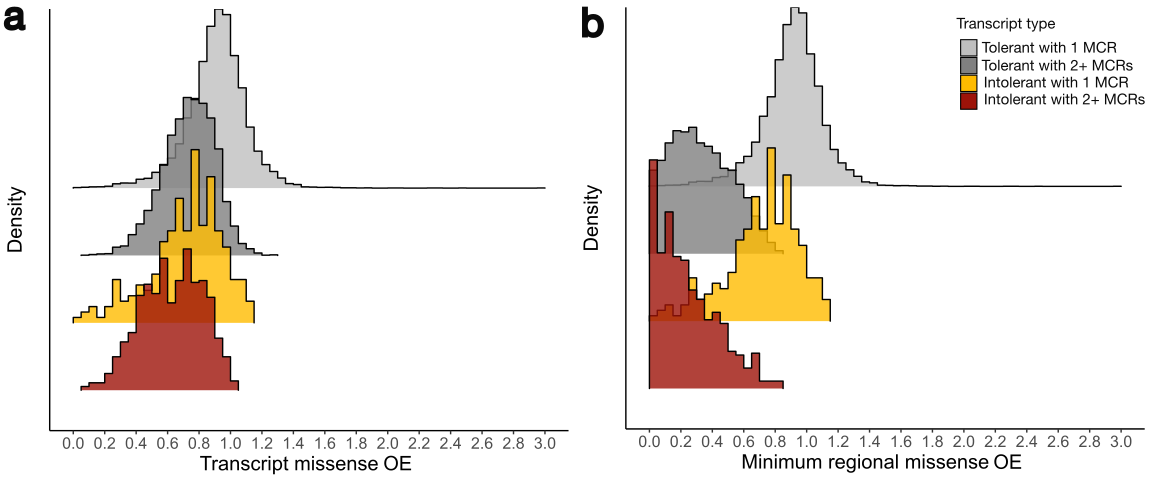


**Supplementary Fig. 4**: Regional missense observed/expected (OE) reveals constraint obscured at the transcript-level.

**a**, The distribution of transcript-wide missense OE across 18,629 transcripts stratified by the combination of two factors: whether the transcript is strongly mutationally intolerant (within first three LOEUF deciles and association with a developmental phenotype in Gene2Phenotype [G2P][^12^](https://paperpile.com/c/UaG5kk/Mn7qq)) and whether we detect multiple missense constraint regions (MCRs). Number of transcripts in each category are: strongly intolerant with multiple MCRs (n=581; red), strongly intolerant with one MCR (n=149; yellow), not strongly intolerant with multiple MCRs (n=4,546; dark gray), not strongly intolerant with one MCR (n=13,353; light gray). **b**, Minimum MCR-level OE using the same groupings.


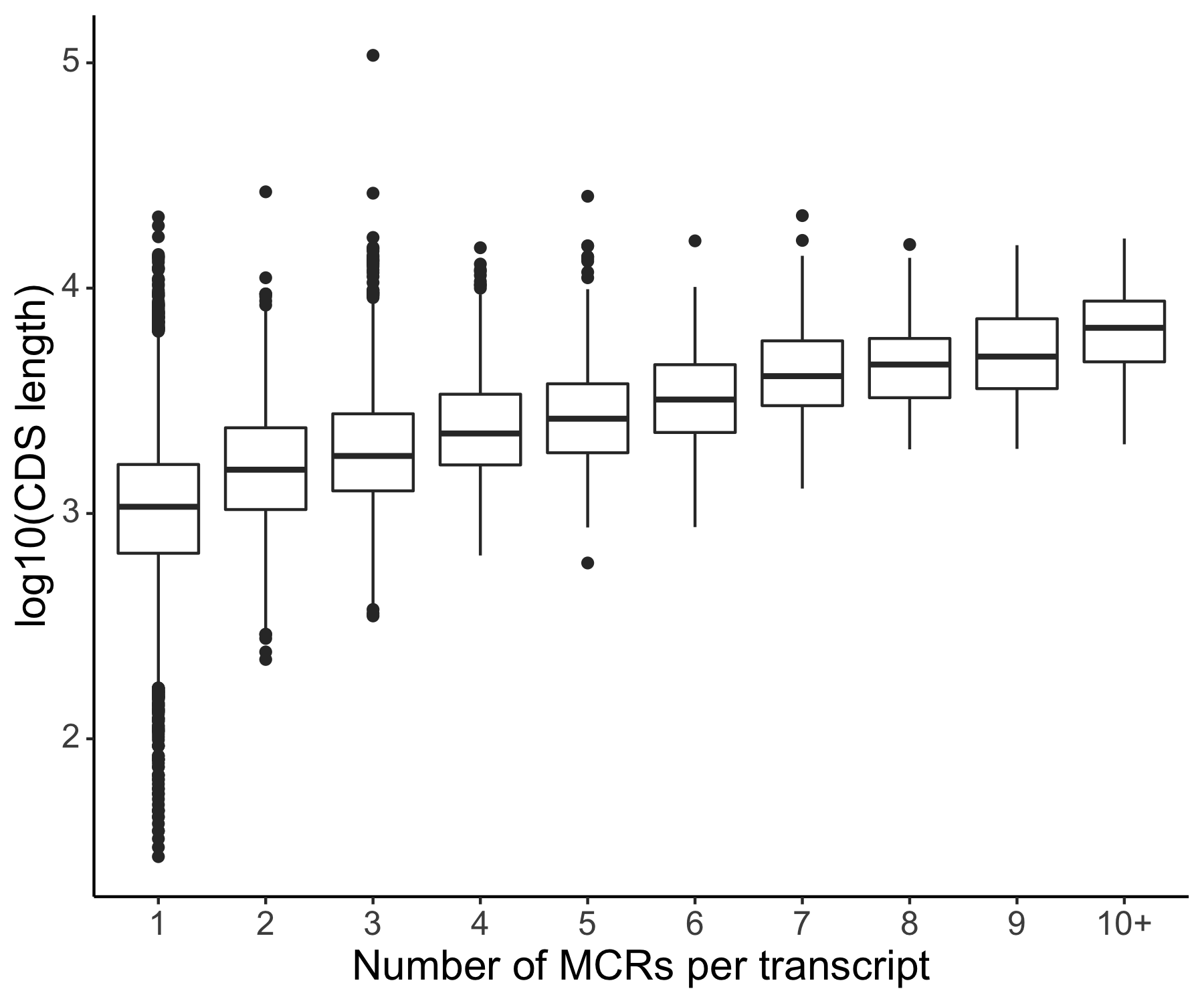


**Supplementary Fig. 5**: Transcript length is correlated with missense constraint region (MCR) count. Coding sequence (CDS) length is measured in base pairs.


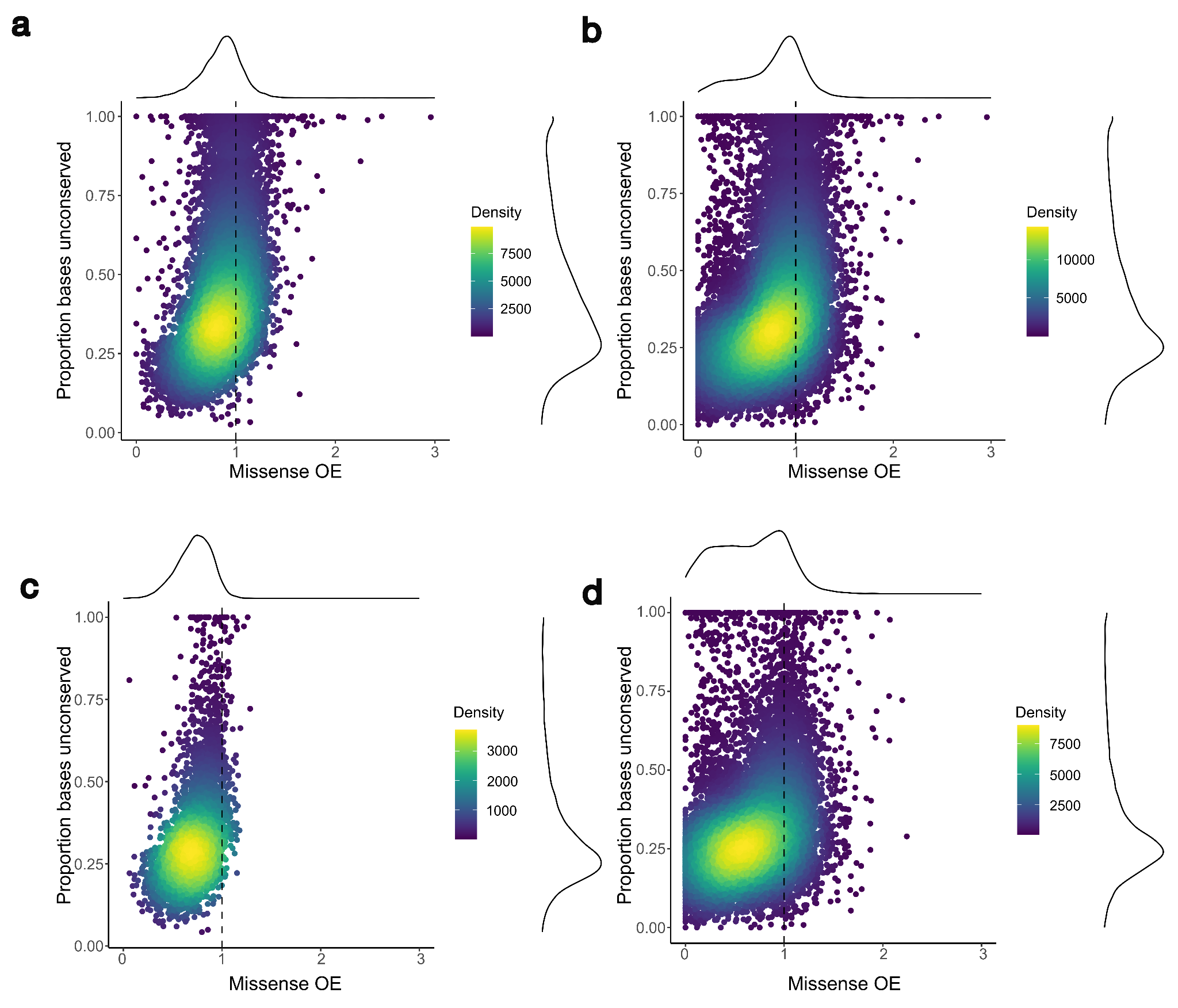


**Supplementary Fig. 6**: Divergence of missense constraint and evolutionary conservation suggests human-specific selective pressures.

Missense observed/expected (OE) vs. proportion of coding bases that are unconserved (phyloP threshold < 2.7)[^6^](https://paperpile.com/c/UaG5kk/1CJ4I) across **a**, entire transcripts (n = 18,629; Spearman ρ = 0.56; p < 10^-50^), **b**, missense constraint regions (MCRs) in these 18,629 transcripts (Spearman ρ = 0.54; p < 10^-50^), **c**, entire transcripts (transcripts with two or more MCRs only; n = 5,127; Spearman ρ = 0.58; p < 10^-50^), and **d**, MCRs in these 5,127 transcripts (Spearman ρ = 0.44; p < 10^-50^). Lighter colors indicate greater density of points.


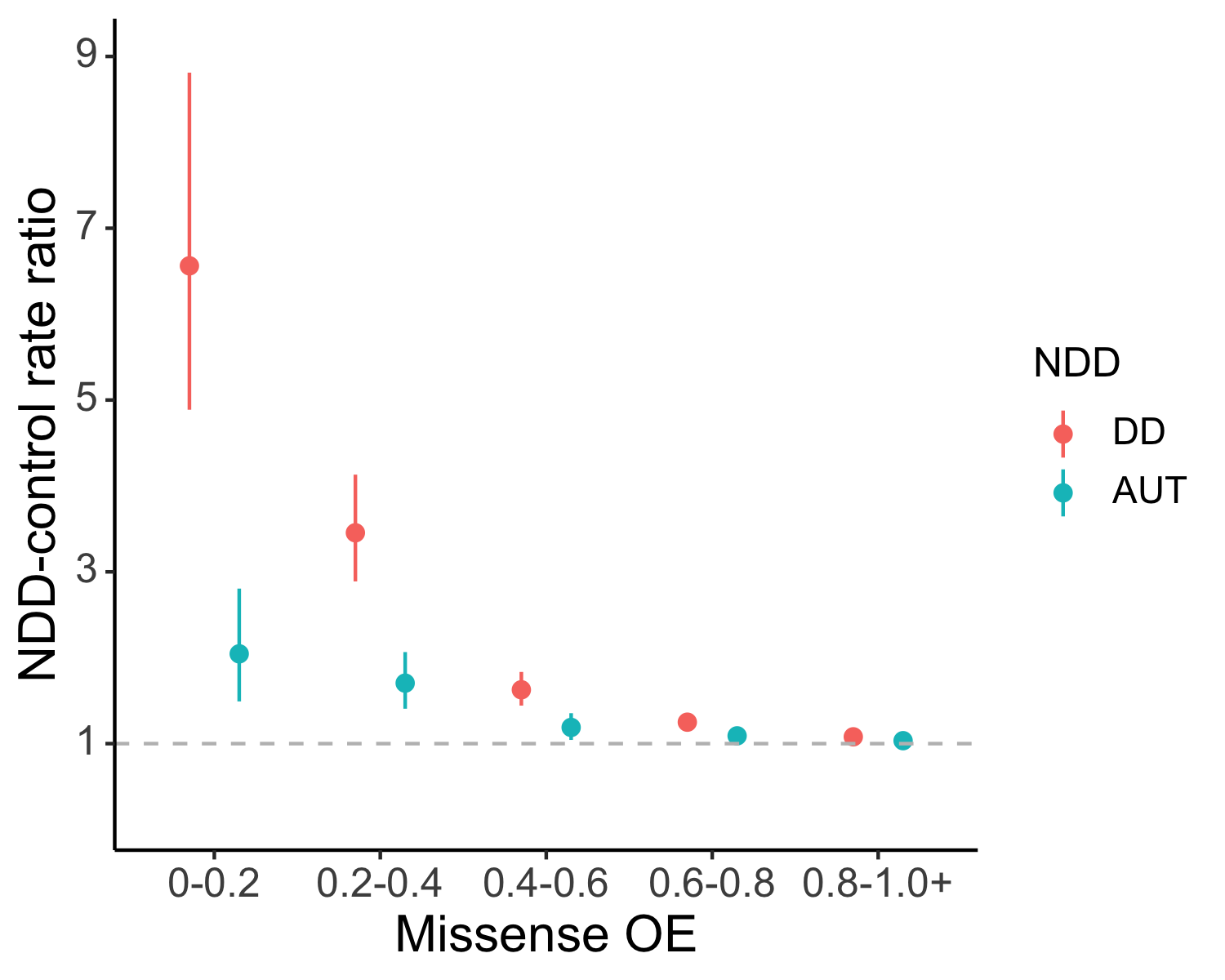


**Supplementary Fig. 7**: Rate ratios of *de novo* missense variants over missense constraint region (MCR) observed/expected (OE) bins in individuals with developmental disorders (DD; red) or autism (AUT; blue) relative to unaffected siblings.

**
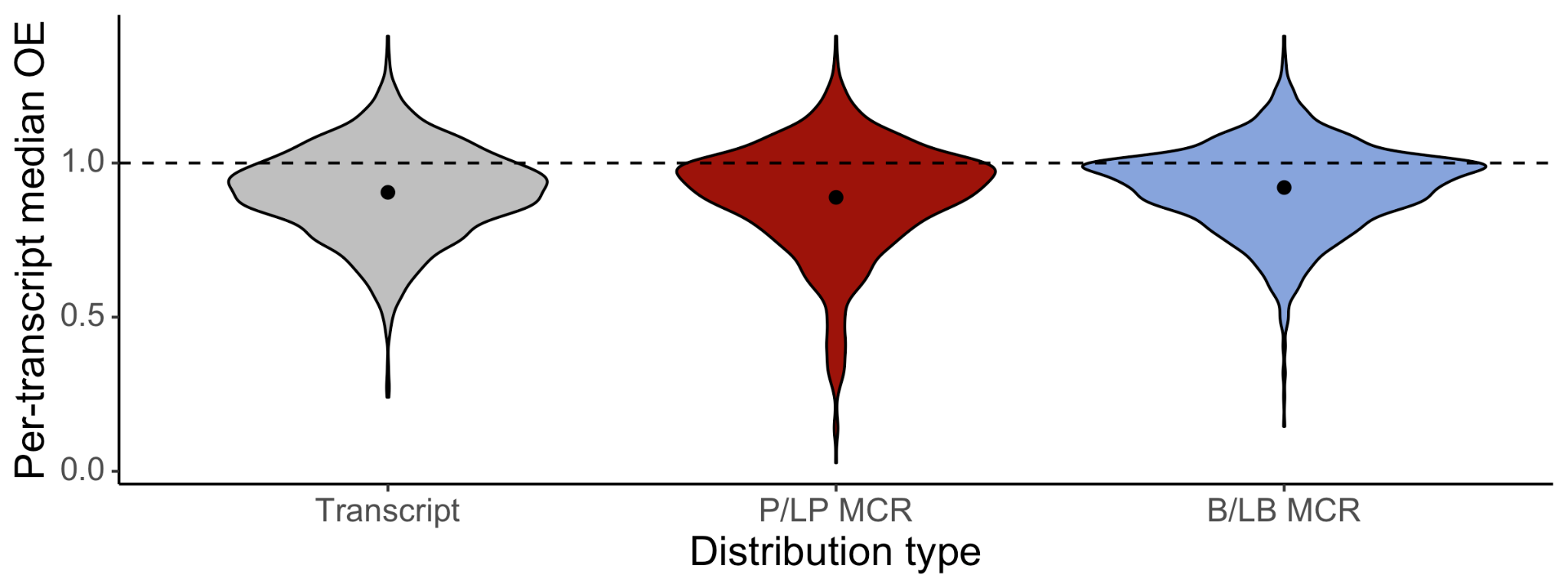
**

**Supplementary Fig. 8**: ClinVar pathogenic/likely pathogenic (P/LP) vs. benign/likely benign (B/LB) localization across missense observed/expected (OE) in autosomal recessive genes.

The distribution within genes with autosomal recessive disease associations of transcript-wide missense OE (gray) and missense constraint region (MCR) OE for ClinVar P/LP (red) and B/LB (blue) missense variants. We filtered to 1,396 transcripts with at least one each of P/LP and B/LB missense variants. For the P/LP and B/LB distributions, we annotated each variant with the missense OE across the MCR they fell in and collapsed these values within each transcript by taking the respective medians.

**
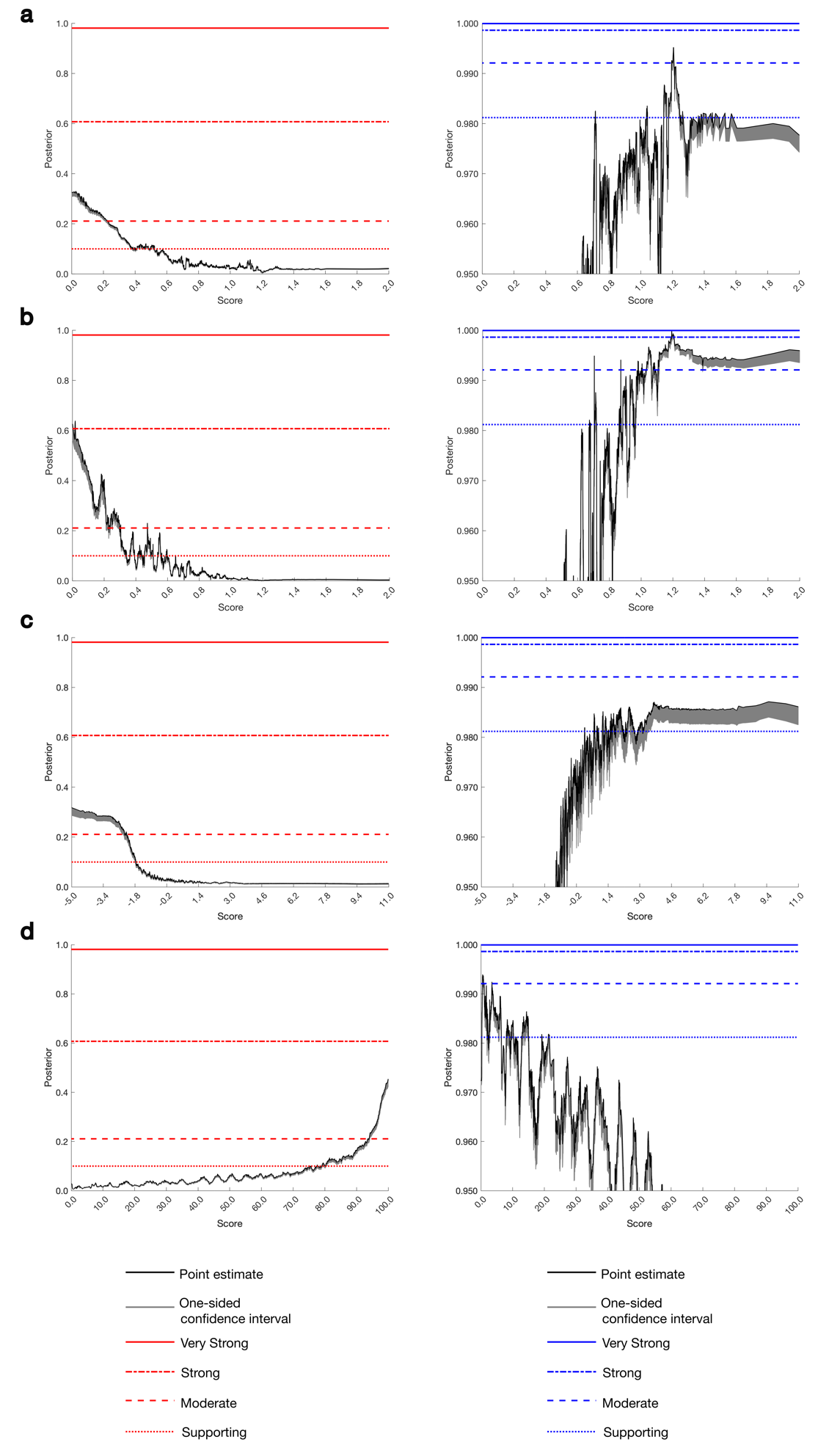
**

**Supplementary Fig. 9**: Clinical calibration of regional constraint tools.

Clinical calibration for pathogenic and benign variation of **a**, Missense constraint region (MCR) missense observed/expected (OE); **b**, MCR missense OE for transcripts with 2+ MCRs only; **c**, COntact Set MISsense tolerance (COSMIS)[^10^](https://paperpile.com/c/UaG5kk/5SNQ) **d**, Constrained coding regions (CCRs)[^11^](https://paperpile.com/c/UaG5kk/auC5). Horizontal lines indicate thresholds required to meet ACMG/AMP evidence levels.

**
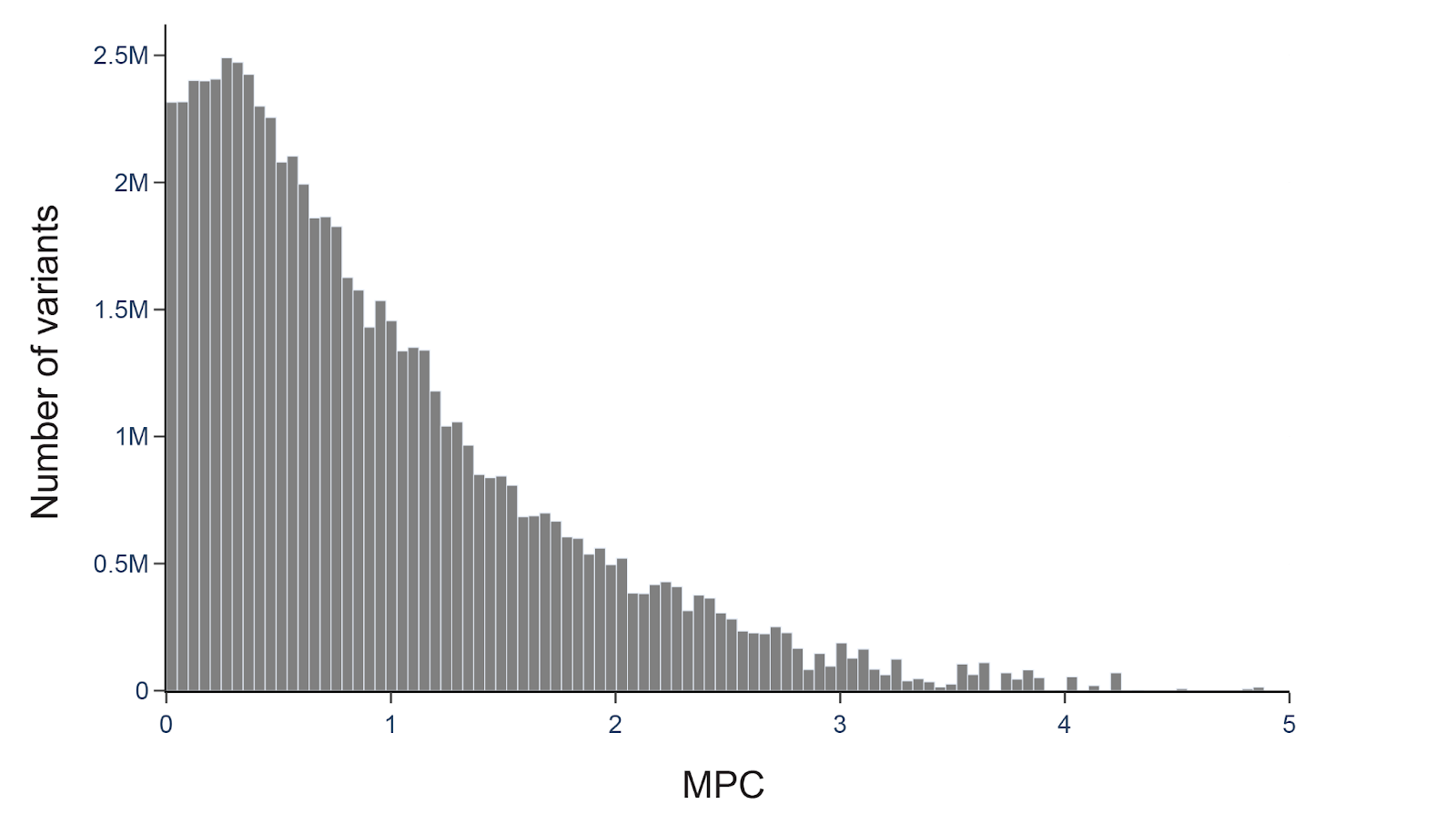
**

**Supplementary Fig. 10**: MPC distribution over missense variants in 18,629 canonical transcripts.


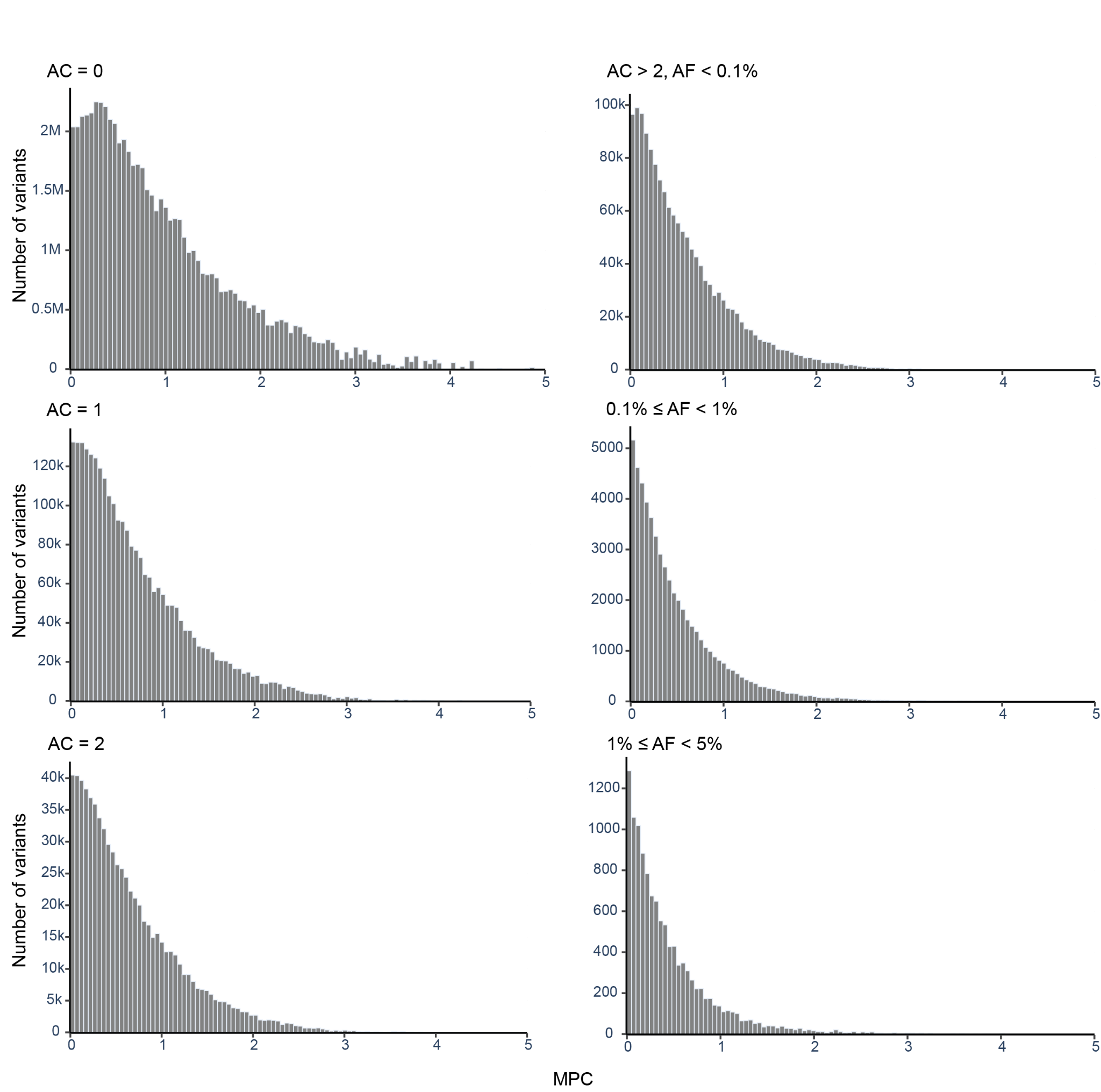


**Supplementary Fig. 11**: MPC distribution over missense variants by gnomAD allele frequency.

**
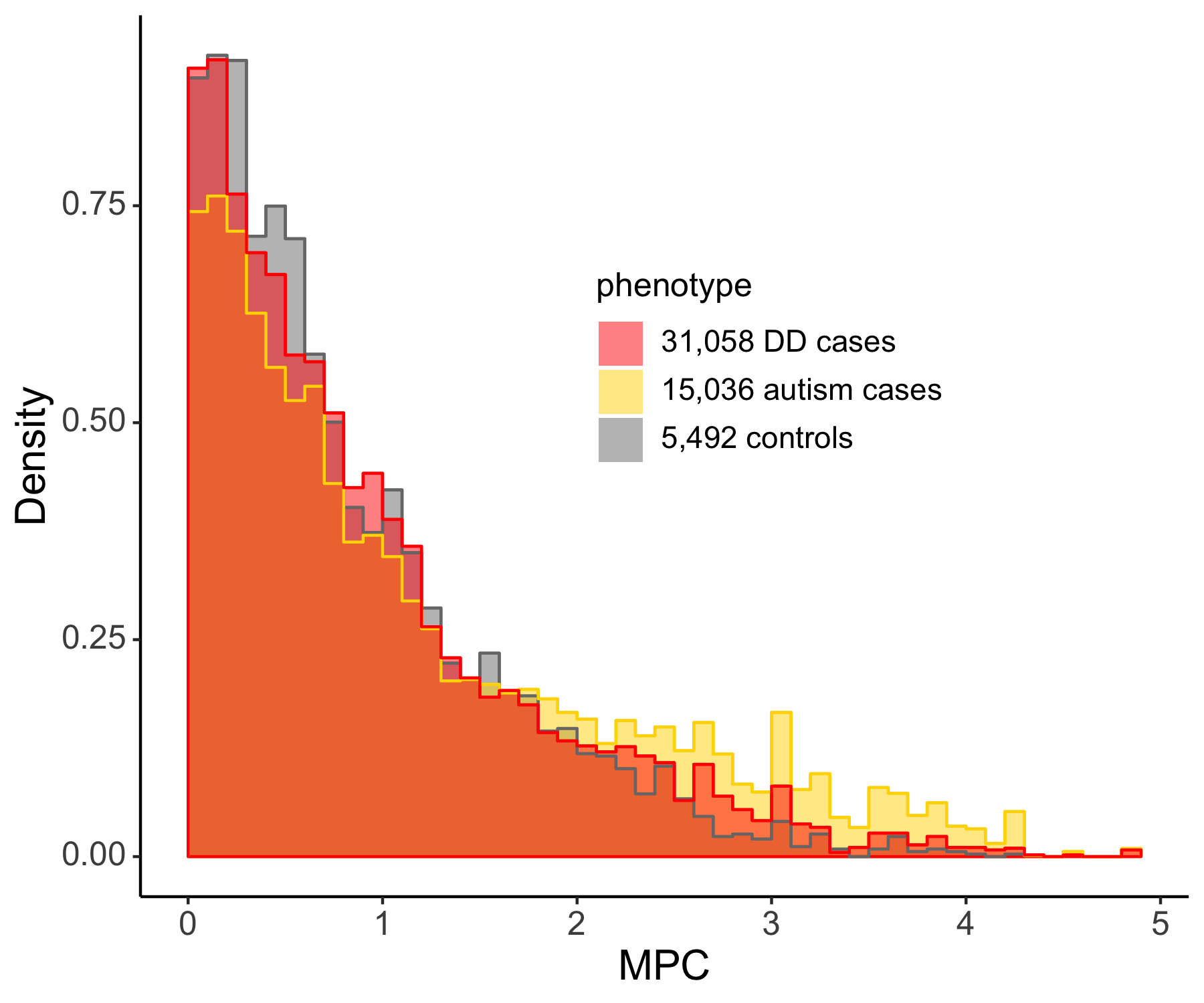
**

**Supplementary Fig. 12**: MPC distribution for *de novo* missense variants in developmental disorder (DD), autistic (AUT), and unaffected sibling cohorts.

MPC scores for DD and AUT *de novo* missense variants are significantly higher than in unaffected siblings (Wilcoxon p < 10^-50^ and = 3.1x10^-5^, respectively). MPC distribution medians are 0.82, 0.68, and 0.61 for DD, AUT, and unaffected siblings, respectively.


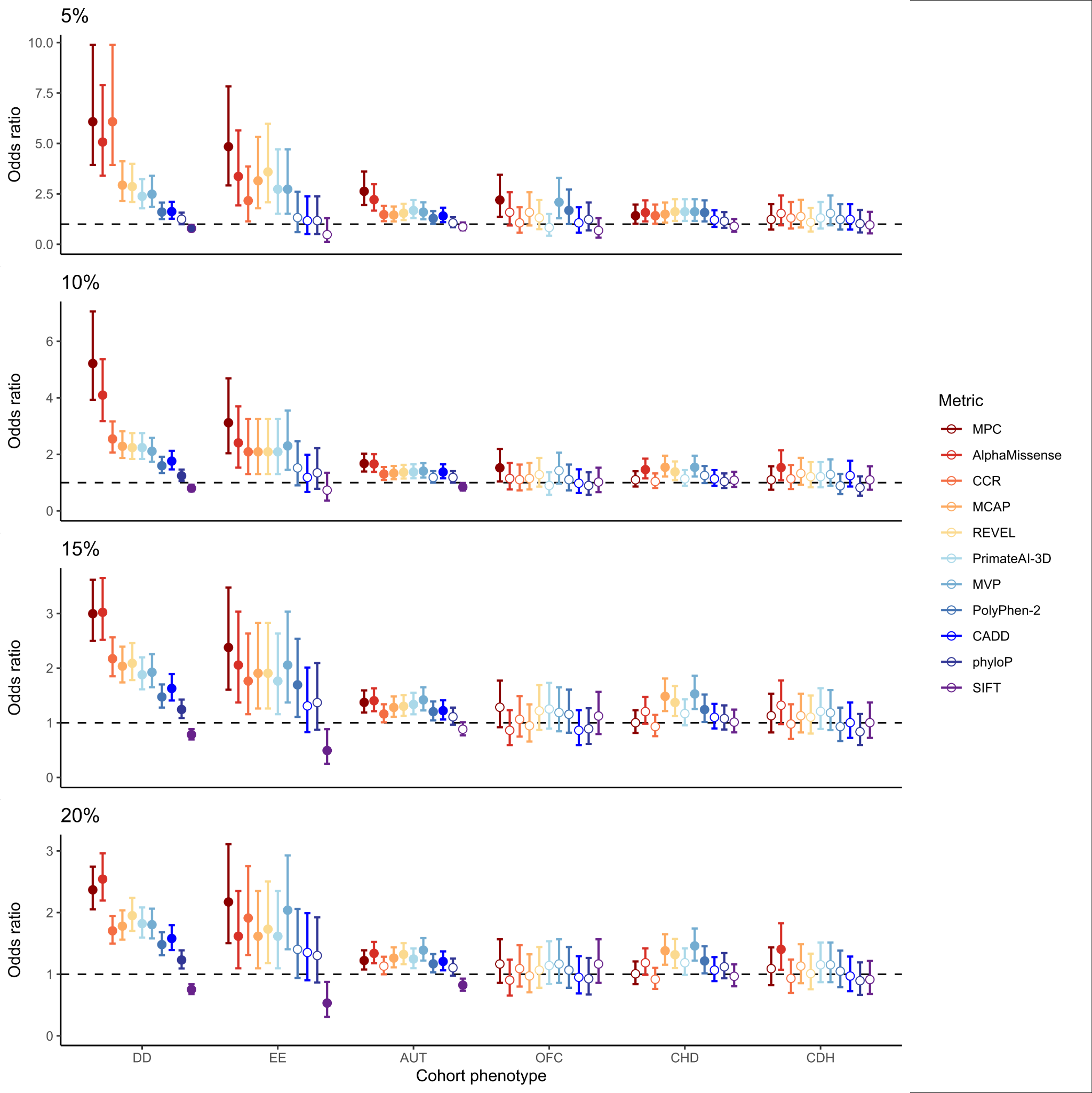


**Supplementary Fig. 13**: MPC stratifies case and control variation at different thresholds. The odds ratio of case to control *de novo* missense variants in the top 5, 10, 15, and 20% of respective rankings. *De novo* missense variants from each case cohort are ranked against those in the 5,492 controls for each predictor. DD: developmental disorders, EE: epileptic encephalopathy, AUT: autism, OFC: orofacial cleft, CHD: congenital heart disease, CDH: congenital diaphragmatic hernia. Error bars represent 95% confidence intervals. Only variants scored by all predictors are included. Points are solid colored if the difference from 1 is statistically significant (binomial or Fisher exact p < 0.05).


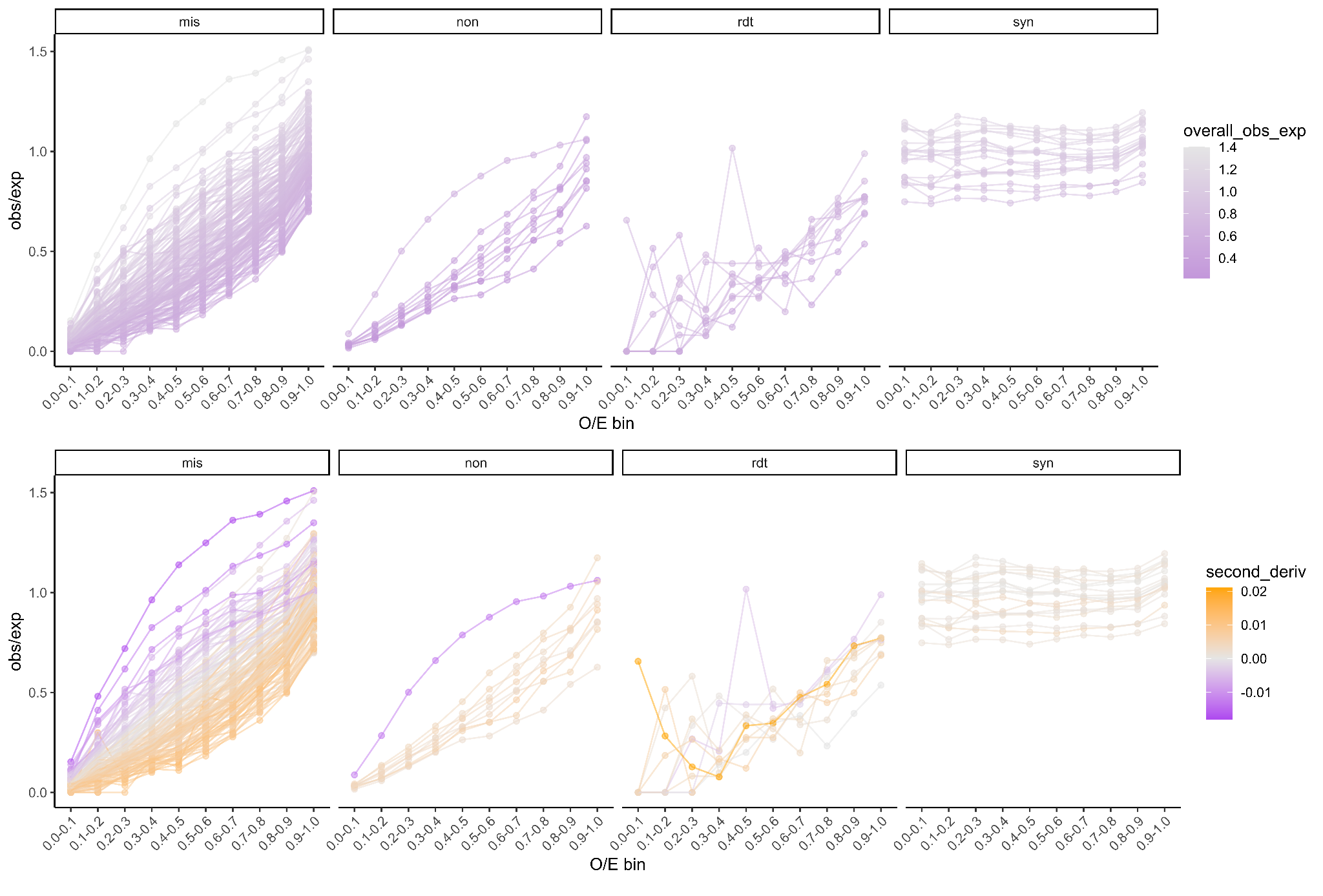


**Supplementary Fig. 14:** Missense constraint region (MCR) observed/expected (OE)-based metrics measuring the increased deleteriousness of amino acid substitution classes that were incorporated into MPC: the overall OE for each substitution and the second derivative of the OE value per OE bin of missense constraint. “mis” = missense; “non” = nonsense; “rdt” = read-through; “syn” = synonymous.

## Additional references in Supplementary Note

1. [Cummings, B. B. *et al.* Transcript expression-aware annotation improves rare variant interpretation. *Nature* **581**, 452–458 (2020).](http://paperpile.com/b/UaG5kk/LKMr)

2. [Kaplanis, J. *et al.* Evidence for 28 genetic disorders discovered by combining healthcare and research data. *Nature* **586**, 757–762 (2020).](http://paperpile.com/b/UaG5kk/86n1)

3. [Fu, J. M. *et al.* Rare coding variation provides insight into the genetic architecture and phenotypic context of autism. *Nat. Genet.* **54**, 1320–1331 (2022).](http://paperpile.com/b/UaG5kk/jRYNZ)

4. [Pejaver, V. *et al.* Calibration of computational tools for missense variant pathogenicity classification and ClinGen recommendations for PP3/BP4 criteria. *Am. J. Hum. Genet.* **109**, 2163–2177 (2022).](http://paperpile.com/b/UaG5kk/4VI4)

5. [Hamosh, A., Scott, A. F., Amberger, J. S., Bocchini, C. A. & McKusick, V. A. Online Mendelian Inheritance in Man (OMIM), a knowledgebase of human genes and genetic disorders. *Nucleic Acids Res.* **33**, D514–7 (2005).](http://paperpile.com/b/UaG5kk/disZ)

6. [Christmas, M. J. *et al.* Evolutionary constraint and innovation across hundreds of placental mammals. *Science* **380**, eabn3943 (2023).](http://paperpile.com/b/UaG5kk/1CJ4I)

7. [Ge, S. X., Jung, D. & Yao, R. ShinyGO: a graphical gene-set enrichment tool for animals and plants. *Bioinformatics* **36**, 2628–2629 (2020).](http://paperpile.com/b/UaG5kk/170e)

8. [Finucane, H. K. *et al.* Partitioning heritability by functional annotation using genome-wide association summary statistics. *Nat. Genet.* **47**, 1228–1235 (2015).](http://paperpile.com/b/UaG5kk/Dw1Y)

9. [Karczewski, K. J. *et al.* The mutational constraint spectrum quantified from variation in 141,456 humans. *Nature* **581**, 434–443 (2020).](http://paperpile.com/b/UaG5kk/h5rG)

10. [Li, B., Roden, D. M. & Capra, J. A. The 3D mutational constraint on amino acid sites in the human proteome. *Nat. Commun.* **13**, 3273 (2022).](http://paperpile.com/b/UaG5kk/5SNQ)

11. [Havrilla, J. M., Pedersen, B. S., Layer, R. M. & Quinlan, A. R. A map of constrained coding regions in the human genome. *Nat. Genet.* **51**, 88–95 (2019).](http://paperpile.com/b/UaG5kk/auC5)

12. [Thormann, A. *et al.* Flexible and scalable diagnostic filtering of genomic variants using G2P with Ensembl VEP. *Nat. Commun.* **10**, 2373 (2019).](http://paperpile.com/b/UaG5kk/Mn7qq)
